# Effect of Leucovorin on Mitochondrial Respiration and One-Carbon Metabolism in Autism Spectrum Disorder: A Double-Blind Placebo-Controlled Substudy

**DOI:** 10.3390/ijms27209101

**Published:** 2026-10-14

**Authors:** Shannon Rose, Stepan Melnyk, S. Jill James, Richard E. Frye

**Affiliations:** 1 College of Nursing, The University of Tennessee Health Science Center, Memphis, TN 38163, USA; srose37@uthsc.edu; 2 Arkansas Children’s Research Institute, Little Rock, AR 72202, USA; sbmelnyk61@gmail.com (S.M.); jamesjill4444@gmail.com (S.J.J.); 3 Department of Pediatrics, University of Arkansas for Medical Sciences, Little Rock, AR 72205, USA; 4 Autism Discovery and Treatment Foundation, Phoenix, AZ 85050, USA

**Keywords:** autism spectrum disorder, leucovorin, folinic acid, mitochondrial respiration, one-carbon metabolism, methylation, S-adenosylmethionine, neurodevelopmental regression, peripheral blood mononuclear cells, biomarker substudy

## Abstract

Mitochondrial dysfunction and impaired methylation are implicated in autism spectrum disorder (ASD). Leucovorin, a reduced folate that bypasses both dihydrofolate reductase and folate receptor alpha, has shown clinical benefit in small trials in children with ASD, but the underlying biology is incompletely understood. In this pre-specified biomarker substudy of a completed double-blind placebo-controlled trial (NCT02000284; NCT01602016), children with ASD received leucovorin (2 mg/kg/day, maximum 50 mg/day) or a matched placebo for 12 weeks. Peripheral blood mononuclear cell respiration was measured by extracellular flux with a graded 2,3-dimethoxy-1,4-naphthoquinone (DMNQ) challenge (38 participants; 17 leucovorin, 21 placebo) and plasma thiol-methylation metabolites were measured pre- and post-treatment (42 participants; 18 leucovorin, 24 placebo). Leucovorin increased basal, ATP-linked, proton-leak, maximal, and reserve respiration relative to the placebo (Group × Visit *p* ≤ 0.012, all q ≤ 0.012; Cohen’s d 0.48–0.92); there was no evidence that the treatment effect varied across the tested DMNQ concentrations. In exploratory moderator analyses, folate receptor alpha autoantibody (FRAA) status modified the effect on maximal respiratory capacity and reserve capacity (both *p* < 0.001), with the largest gains in FRAA-positive children receiving leucovorin, and a history of neurodevelopmental regression modified the effect on basal, proton-leak and reserve respiration. The plasma SAM/SAH ratio increased (d = 0.77) and S-adenosylhomocysteine decreased (d = −0.67) on leucovorin; both were nominally significant (*p* = 0.020 and 0.050) but neither survived false-discovery-rate correction within the thiol family. The findings support the folate–mitochondrial–redox axis as a candidate therapeutic target in ASD and warrant replication in larger independent cohorts.

## 1. Introduction

Autism spectrum disorder (ASD) is a neurodevelopmental condition characterized by social communication differences and restricted or repetitive behaviors [1]. A substantial subset of children with ASD show biomarkers of mitochondrial dysfunction [2]. Systematic reviews estimate that 5–80% of individuals with ASD show at least one biomarker of mitochondrial dysfunction depending on the marker used, while approximately 5% meet the criteria for mitochondrial disease compared with a population prevalence of mitochondrial disease below 0.1% [3]. Abnormalities in mitochondrial respiration, electron transport chain (ETC) function and oxidative stress markers have been reported in ASD across the brain, muscle and peripheral blood, although the specificity of these findings to ASD remains uncertain and comparable abnormalities are seen in other neurodevelopmental and neuropsychiatric conditions [3].

Folate metabolism is closely linked to mitochondrial function through one-carbon metabolism (OCM), which supplies substrates for nucleotide synthesis, methylation reactions and redox homeostasis [4]. Leucovorin (folinic acid), a reduced form of folate that bypasses dihydrofolate reductase, has been proposed as a candidate therapy because it can raise cerebrospinal fluid folate when folate receptor alpha (FRα)-mediated transport is impaired. A double-blind placebo-controlled (DBPC) trial in children with ASD and language impairment reported that leucovorin (2 mg/kg/day, maximum 50 mg/day) improved verbal communication [5]. Supportive findings have been reported in three additional small randomized controlled trials that examined different symptom domains and outcome scales [6,7,8]. Overall, the available evidence base is limited to a small number of short-duration randomized trials with heterogeneous outcome measures, and larger confirmatory trials are needed. The need for caution is reinforced by the retraction of a 2024 randomized trial of oral folinic acid in ASD [9]. The retraction noted errors in the reported tables and concerns regarding the statistical analyses with a post-publication statistical review unable to reproduce the results [9]. Thus, herein we present both unadjusted and false-discovery-rate-adjusted inference and pre-specified sensitivity analyses for every primary claim.

The methionine cycle produces metabolites that reflect cellular methylation capacity. S-adenosylmethionine (SAM) is the universal methyl donor and S-adenosylhomocysteine (SAH) is the product of methyl transfer; the SAM/SAH ratio indexes methylation potential [4]. Homocysteine is either remethylated to methionine via folate-dependent pathways or diverted to trans-sulfuration, generating cysteine and glutathione. Children with ASD have been reported to show decreased SAM/SAH ratios [10] and other thiol abnormalities, particularly when mitochondrial dysfunction is also present [11].

Here, we report an exploratory biomarker substudy of the completed DBPC leucovorin trial [5]. We examined the association between leucovorin treatment and (a) PBMC mitochondrial respiration measured at baseline and under graded in vitro oxidative stress and (b) plasma methylation-cycle metabolites. Subgroup analyses examined clinical response on verbal communication and the history of neurodevelopmental disorder (NDR) as potential moderators. Given the modest sample size and the number of statistical models tested, the findings are framed throughout as hypothesis-generating and requiring replication.

## 2. Results

### 2.1. Participant Characteristics

Age, sex distribution and prevalence of a history of NDR did not differ significantly between the leucovorin and placebo groups (Table 1). The clinical responder rate on verbal communication was higher in the leucovorin-treated group than in the placebo group, as previously reported for the parent trial [5].

The relationship between clinical response and NDR was examined to check for confounding of the subgroup analyses. Overall, the clinical response rate did not differ by NDR history (Fisher’s exact test: OR = 0.42, *p* = 0.323). When stratified by treatment group, the leucovorin group showed no difference in NDR rates between responders (40.0%) and non-responders (42.9%; Fisher’s exact *p* = 1.000). The Cochran–Mantel–Haenszel test, controlling for the treatment group, confirmed the absence of a significant association between NDR and responder status (χ^2^ = 1.26, df = 1, *p* = 0.261; common OR = 0.44). Logistic regression confirmed that the treatment group, but not NDR, was a significant predictor of responder status (OR = 4.80, 95% CI 1.16–19.75, *p* = 0.030).

### 2.2. Primary Analysis: Effect of Leucovorin on Mitochondrial Respiration

#### 2.2.1. Overall Effect

**Figure 1:**
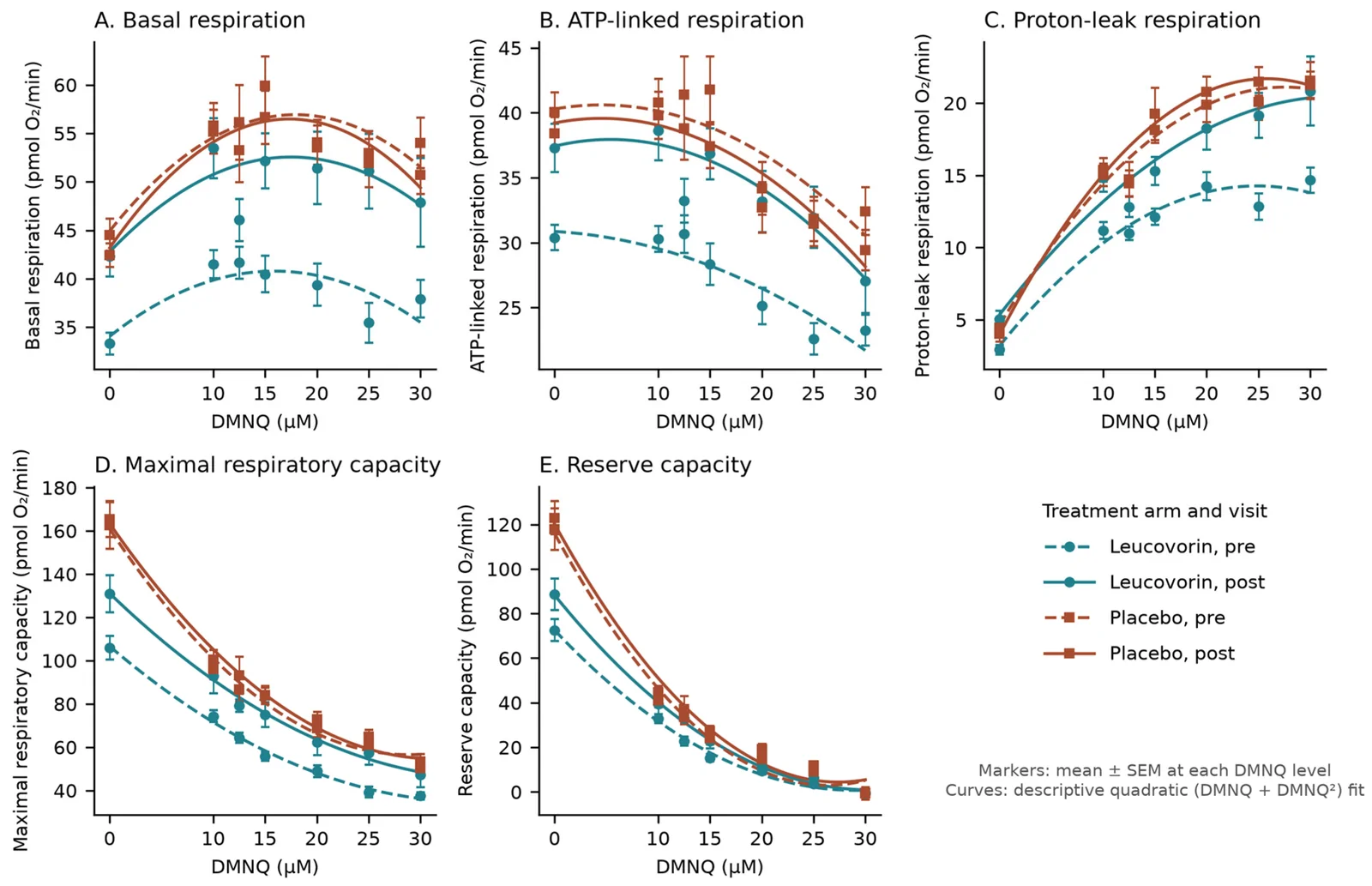
DMNQ dose-response curves for mitochondrial respiratory parameters by treatment arm and visit. Each panel shows the relationship between DMNQ concentration (µM) and the indicated respiratory parameter at Visit 1 (pre-treatment, dashed) and Visit 2 (post-treatment, solid) for the leucovorin (teal) and placebo (rust) arms. Panels are labeled (**A**–**E**). Symbols are arm means at each DMNQ concentration and error bars are standard errors of the mean; curves are descriptive quadratic (DMNQ + DMNQ^2^) least-square fits to the well-level observations of the same series, shown to display the non-monotonic shape of the dose-response and carrying no inferential weight. Respiration is expressed as pmol O_2_/min per well (4 × 10^5^ PBMCs seeded). *n* = 38 participants (17 leucovorin, 21 placebo) contributing 1805 well-level observations after source-data verification. Inferential estimates are from the mixed-effects model and are reported in Table 2 and Supplementary Table S1, not read from this figure.

The primary result is reported in Table 2. Relative to the placebo, leucovorin increased all five respiratory parameters from pre- to post-treatment: BR (Cohen’s d = 0.81, 95% CI 0.46–1.17), ALR (d = 0.84, 0.49–1.18), PLR (d = 0.48, 0.11–0.85), MRC (d = 0.92, 0.57–1.26) and RC (d = 0.71, 0.35–1.08). All five remained significant after Benjamini–Hochberg correction across the respiratory family (all q ≤ 0.012; Table 2; Supplementary Table S1; Figure 1). Effect sizes are computed as β/√(σ^2^_subject + σ^2^_residual) from the final model, and the convention is documented in the Supplementary Tables; full unadjusted *p*-values, Benjamini–Hochberg q-values, and 95% confidence intervals for all effect sizes are provided there.

There was no evidence that the treatment effect varied across the tested DMNQ range: the Group × Visit × DMNQ and Group × Visit × DMNQ^2^ interactions were not significant for any respiratory parameter (all *p* ≥ 0.17; Supplementary Table S1). The absence of a detectable interaction does not demonstrate that the effect was equivalent across the dose range; the study was not powered to detect an interaction of this kind. Independent of treatment, the groups differed in the shape of the DMNQ dose-response for MRC and RC (Group × DMNQ and Group × DMNQ^2^, all *p* ≤ 0.002), a baseline difference in stress response rather than a treatment effect. The groups also differed in level at baseline: at DMNQ 0 and Visit 1, participants randomized to the placebo had higher BR (+12.80, *p* = 0.007), ALR (+11.07, *p* = 0.002), MRC (+58.93, *p* < 0.001) and RC (+46.08, *p* < 0.001) than those randomized to leucovorin, with no difference in PLR (+1.75, *p* = 0.343; Supplementary Table S1). Allocation was randomized, so this imbalance arose by chance rather than through selection. The primary contrast compares within-participant pre-to-post change between arms, which removes the additive effect of the differing starting levels, but it does not by itself render the estimate immune to them: where one arm begins further from the cohort mean, part of its subsequent movement may reflect regression to the mean, and with 17 and 21 participants, that component cannot be cleanly separated from treatment. The two arms therefore began from materially different respiratory set-points; that difference contributes to the baseline-dependent subgroup patterns described in Section 2.2.2, and the possibility that it also contributes to the magnitude of the primary contrast is acknowledged in Section 3.4.

#### 2.2.2. Effect of Clinical Response

**Figure 2:**
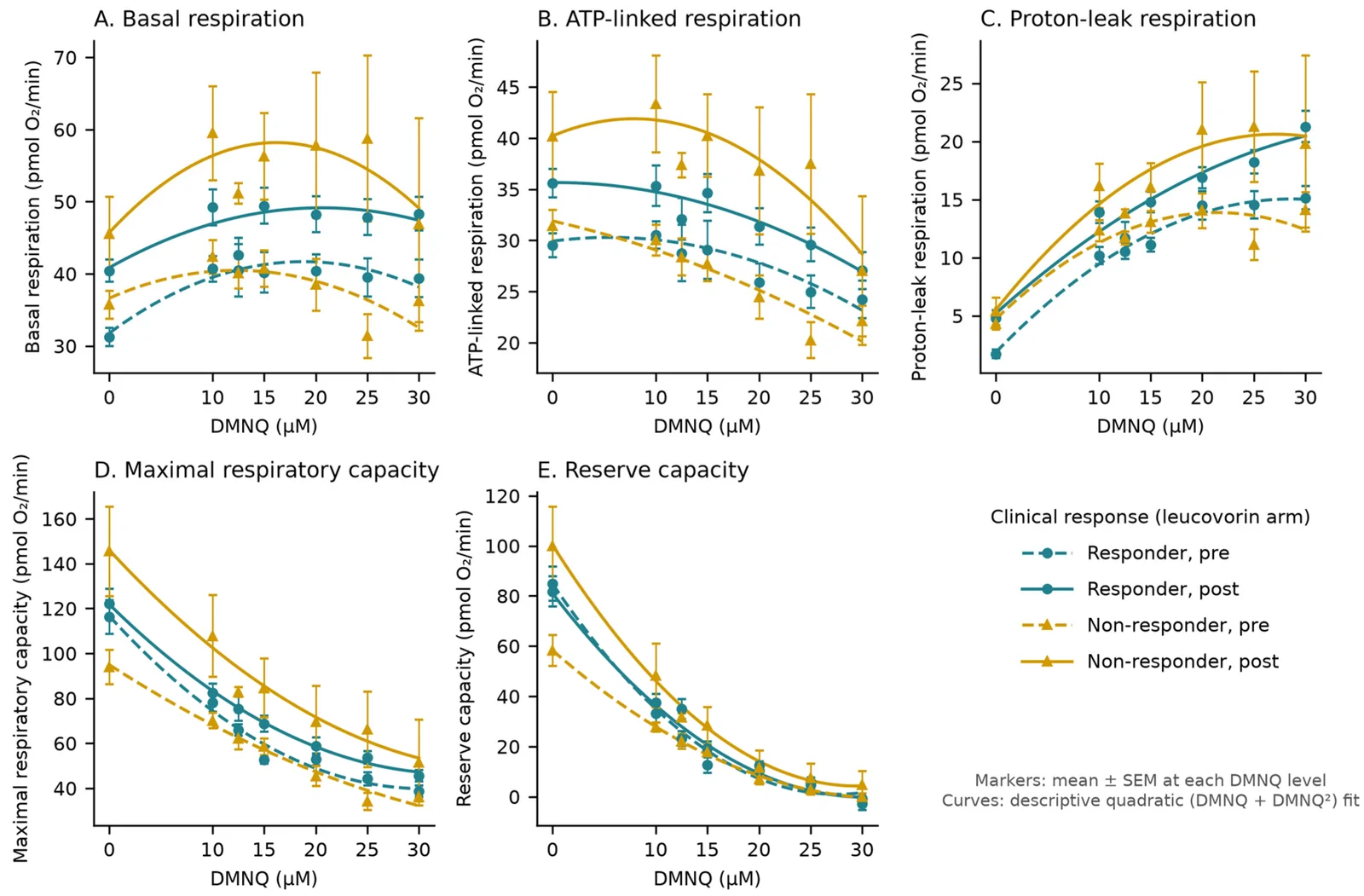
DMNQ dose-response curves comparing clinical responders and non-responders within the leucovorin arm. Responder status is the parent trial’s pre-specified designation, a five-standardized-point increase in verbal communication on the ability-appropriate primary instrument [5]. Panels, symbols, error bars and fitted curves are as seen in Figure 1 (responders teal, non-responders gold; dashed = Visit 1, solid = Visit 2); *n* = 10 responders and 7 non-responders. Responders entered treatment with higher maximal and reserve capacity than non-responders, which is relevant to interpreting the within-arm change (Section 2.2.2). Cell sizes are small, and these curves illustrate an exploratory subgroup pattern only.

Within the leucovorin arm at baseline, BR, ALR and PLR did not differ significantly between clinical responders and non-responders (all *p* ≥ 0.23), whereas responders had higher MRC (d = +0.78, *p* = 0.018) and RC (d = +1.16, *p* < 0.001) than non-responders (Figure 2; Supplementary Table S2). The corresponding contrasts in the placebo arm ran in the opposite direction (MRC d = −1.32, *p* = 0.008; RC d = −1.40, *p* = 0.002), so the baseline responder profile is specific to the leucovorin arm and is reported separately for each arm in Supplementary Table S2.

Both responder subgroups within the leucovorin arm increased respiration from pre- to post-treatment, but the pattern differed by parameter rather than uniformly favoring one subgroup. Non-responders showed larger gains in ALR (d = 0.66 versus 0.45), MRC (d = 1.11 versus 0.12), and RC (d = 1.33 versus −0.19), whereas responders showed larger gains in BR (d = 0.58 versus 0.51) and PLR (d = 0.58 versus 0.21). The Respond × Visit interaction within the leucovorin arm was significant for MRC (β = −62.79, *p* < 0.001) and RC (β = −56.77, *p* < 0.001), both indicating smaller post-treatment gains in responders. These are the leucovorin-arm contrasts implied by the full model, obtained by adding the Responder × Visit and Group × Responder × Visit coefficients of Supplementary Table S2; refitting each arm separately gives closely similar values (MRC −62.28, RC −56.24). These contrasts rest on 10 responders and seven non-responders within the leucovorin arm and carry no multiplicity adjustment and are exploratory. Responder status is a post-randomization clinical outcome, so this comparison is descriptive rather than a randomized contrast and cannot support a causal or mechanistic conclusion (Figure 2; Supplementary Table S2).

This subgroup pattern requires explicit interpretation because it is easily read as contradictory. Responders entered treatment with substantially higher MRC and RC than non-responders (d = +0.78 and +1.16; Supplementary Table S2), so non-responders began with considerably more headroom on those two parameters, and the magnitude of change is not directly comparable between the strata. Responders nonetheless showed larger increases in unstressed basal and proton-leak respiration. With no evidence of differential dose-response terms in these data (Section 2.2.1), we do not interpret these subgroups as differing in resilience to oxidative challenge. What remains is a baseline-dependent difference in the magnitude of change, consistent with the high-flux, low-reserve mitochondrial phenotype previously described in a subset of children with ASD [3,12], and a reminder that an increase in respiration is not uniformly favorable. These contrasts are exploratory and unadjusted.

#### 2.2.3. Effect of Neurodevelopmental Regression

**Figure 3:**
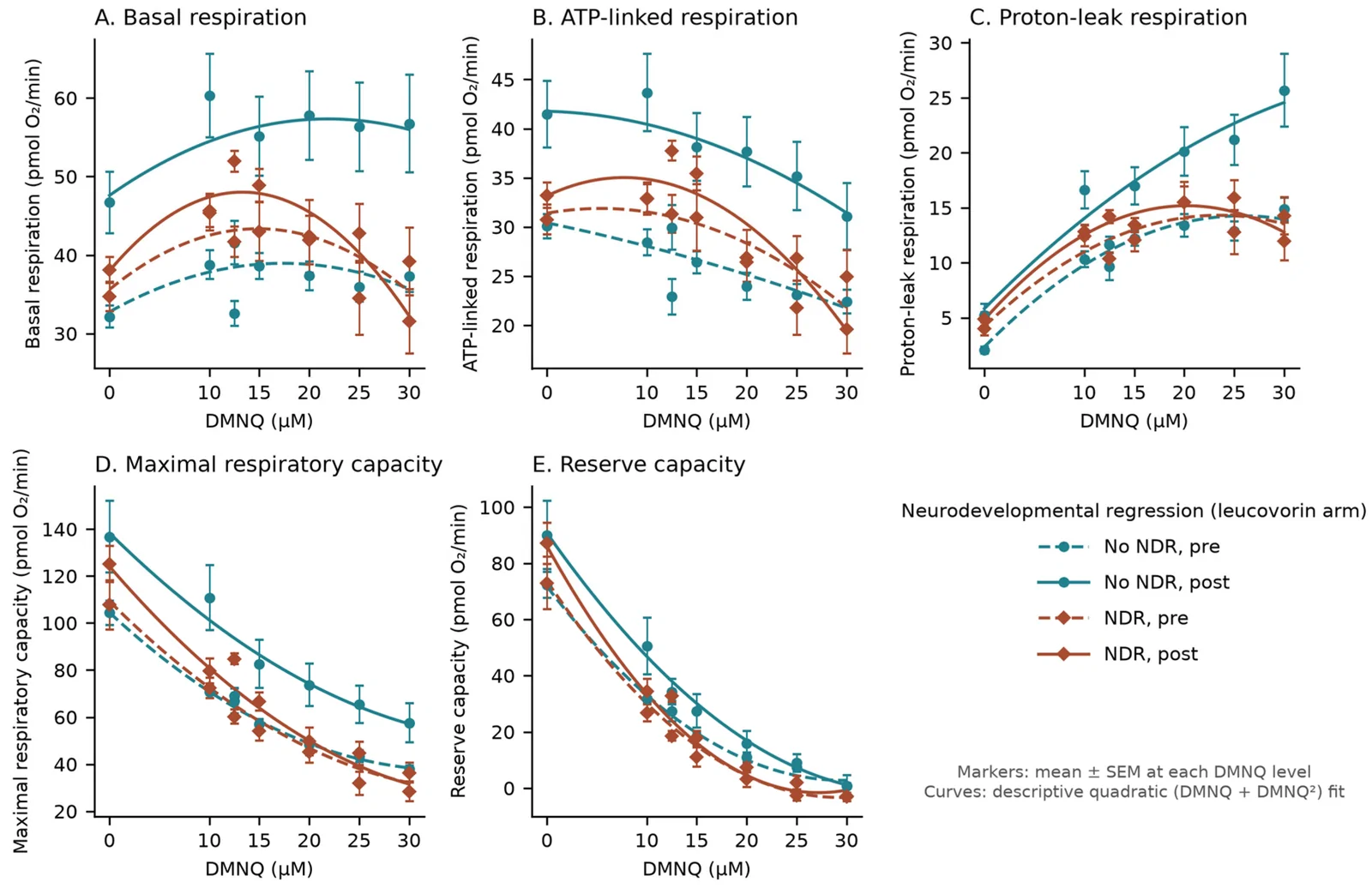
DMNQ dose-response curves for leucovorin-treated children with (rust) and without (teal) a history of neurodevelopmental regression (NDR). Panels, symbols, error bars and fitted curves are as seen in Figure 1 (dashed = Visit 1, solid = Visit 2); *n* = 7 with and 10 without a history of NDR. NDR was classified from the parent report and should be interpreted as an exploratory moderator.

A history of NDR modified the leucovorin effect for three of the five respiratory parameters. The Group × NDR × Visit interaction was significant for BR (β = −10.99, *p* = 0.002), PLR (β = −9.25, *p* < 0.001), and RC (β = +17.44, *p* = 0.008), and was not significant for ALR (*p* = 0.514) or MRC (*p* = 0.446). This coefficient tests whether the NDR-related difference in pre-to-post change differs between the treatment arms; it does not by itself describe the change within either arm. The arm-specific simple effects of the same model are therefore reported in Supplementary Table S3a. Within the leucovorin arm, children without a history of NDR improved on every parameter (BR β = +21.33, 95% CI +15.68 to +26.98; RC β = +28.76, 95% CI +18.46 to +39.05; both *p* < 0.001), whereas those with a history of NDR improved less (BR β = +2.13, *p* = 0.454; RC β = +11.55, 95% CI +1.37 to +21.73, *p* = 0.026). The within-leucovorin NDR contrast was accordingly negative for all five parameters, including reserve capacity (β = −17.21, 95% CI −26.58 to −7.84). The positive sign of the RC interaction therefore does not indicate a larger reserve-capacity gain among leucovorin-treated children with NDR; it arises because NDR history was associated with a steeper reserve-capacity decline under the placebo (β = −34.65, 95% CI −43.41 to −25.90) than the attenuation of gain seen under leucovorin (Figure 3; Supplementary Tables S3 and S3a). Because NDR was classified from the parent report and the subgroups comprise seven leucovorin-treated children with and ten without a history of NDR, this pattern is exploratory.

#### 2.2.4. Folate Receptor Alpha Autoantibody Status

FRAA status was available for all 38 participants in the respirometry cohort (nine of seventeen positive on leucovorin, 14 of 21 on the placebo) and for all 42 in the thiol cohort (26 of 42 positive, 62%; blocking autoantibody, seven; binding autoantibody, 22; Table 1); FRAA prevalence did not differ between arms (Fisher exact *p* = 0.21). FRAA status modified the leucovorin effect for the two stress-dependent respiratory parameters: Group × FRAA × Visit was significant for MRC (β = +38.29, SE 8.13, *p* < 0.001, d = +0.81) and RC (β = +35.73, SE 6.69, *p* < 0.001, d = +0.99) and was not significant for BR (*p* = 0.493), ALR (*p* = 0.056) or PLR (*p* = 0.132). The direction is consistent with the parent trial. The model-estimated arm-specific simple effects at DMNQ 0 are given in Supplementary Table S9, Panel C. Within the leucovorin arm, the pre-to-post change in MRC was +44.41 pmol/min (95% CI +32.04 to +56.77) in FRAA-positive and +16.45 (+3.59 to +29.31) in FRAA-negative participants, a within-arm difference of +27.96 (+16.42 to +39.50); within the placebo arm, it was −15.20 (−27.27 to −3.13) and −4.87 (−18.01 to +8.26), respectively, a difference of −10.33 (−21.34 to +0.68). For RC, the within-leucovorin difference was +22.35 (+12.86 to +31.85) and the within-placebo difference was −13.37 (−22.42 to −4.32). The difference between the two within-arm contrasts equals the Group × FRAA × Visit coefficient in each case (MRC +27.96 − [−10.33] = +38.29; RC +22.35 − [−13.37] = +35.73), so the interaction is carried by both arms moving apart rather than by one arm alone (Supplementary Table S9, Panels A and C).

In the thiol family, which carries Benjamini–Hochberg correction across its five members, FRAA status modified the leucovorin effect on methionine (β = −5.56, *p* = 0.002, q = 0.012, d = −1.85); the effect on the SAM/SAH ratio was nominal and did not survive correction (β = −0.61, *p* = 0.036, q = 0.091, d = −1.36). Within the leucovorin arm, FRAA-negative participants increased methionine (20.85 to 22.38 µmol/L), whereas FRAA-positive participants decreased (21.56 to 20.19 µmol/L); both FRAA strata increased the SAM/SAH ratio on leucovorin (FRAA-negative 2.74 to 3.01, FRAA-positive 2.69 to 2.87), while the placebo strata declined or were static. These analyses are exploratory and based on seven to seventeen participants per cell. Multiplicity is handled differently in the two families, and the distinction matters: the thiol moderator analyses reported here carry false-discovery-rate correction within the five-member thiol family, whereas the respiratory moderator analyses in the preceding paragraph are reported with unadjusted *p*-values and carry no correction across the five respiratory parameters or across the three moderators examined. They are reported because the parent trial identified FRAA status as the strongest predictor of clinical response [5].

#### 2.2.5. Sex and Age

Sex and age were examined as exploratory moderators. Group × Sex × Visit was significant for BR (β = −21.26, *p* < 0.001), ALR (β = −9.92, *p* = 0.005), PLR (β = −11.35, *p* < 0.001) and MRC (β = −30.44, *p* = 0.005) but not for RC (*p* = 0.284). With only eight girls in the cohort, three of them in the leucovorin arm, these coefficients are estimated from very few participants and are not interpretable as evidence of a sex difference in treatment response; they are reported for completeness and to document that the primary analysis was not stratified by sex. Group × Age × Visit was not significant for BR (β = +0.07, *p* = 0.92), ALR (β = +0.42, *p* = 0.35) or PLR (β = −0.36, *p* = 0.19), but was significant for MRC (β = +5.49, *p* < 0.001) and RC (β = +5.38, *p* < 0.001), suggesting that the treatment-associated gain in stress-induced spare respiratory capacity was larger in older children. Both age coefficients correspond to small, standardized effects (d ≤ 0.15), and the age moderation is estimated from a single wide age band rather than from age-stratified enrollment. No thiol outcome was significantly moderated by sex or age (all *p* ≥ 0.072, all q ≥ 0.215; Supplementary Table S9, Panel B). The study was neither designed nor powered to characterize sex or age moderation (Supplementary Table S9, Panel A).

### 2.3. Primary Analysis: Thiol Metabolites and One-Carbon Metabolism

**Figure 4:**
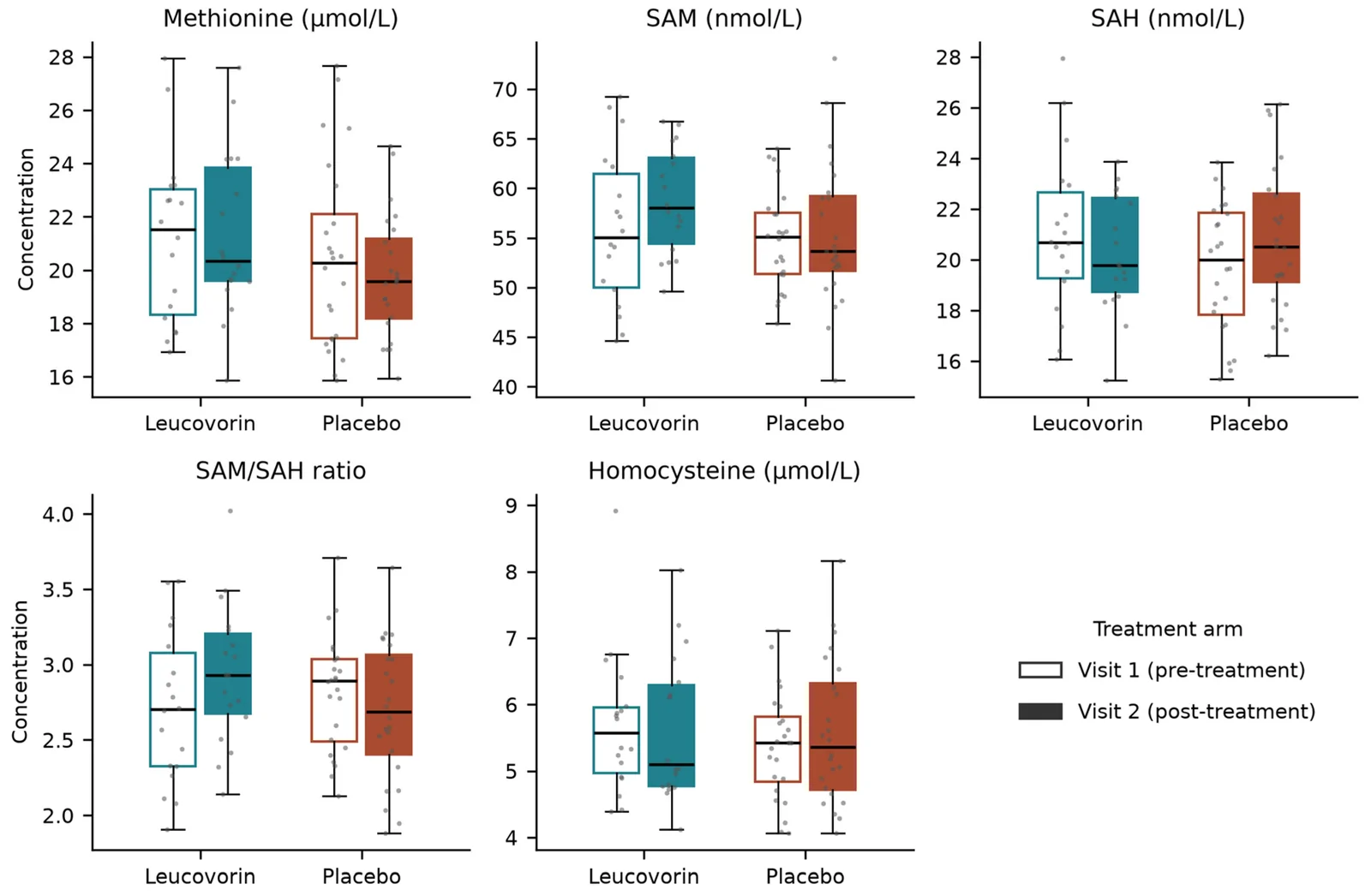
Plasma methylation-cycle metabolites (methionine, SAM, SAH, SAM/SAH ratio, homocysteine) pre- and post-treatment by treatment arm. Box plots show the median and interquartile range with whiskers to 1.5 × IQR; open boxes are Visit 1 (pre-treatment) and filled boxes are Visit 2 (post-treatment). Leucovorin, teal; placebo, rust. Gray points are individual participants (*n* = 42; 18 leucovorin, 24 placebo). Group × Visit estimates, nominal *p*-values and Benjamini–Hochberg q-values are reported in Supplementary Table S4; no metabolite survives correction at q < 0.05.

Two methylation-related metabolites showed a nominally significant effect of leucovorin, neither of which survived Benjamini–Hochberg correction across the five-member thiol family (Figure 4; Supplementary Table S4). The SAM/SAH ratio showed the largest treatment effect (d = 0.77, *p* = 0.020, q = 0.098): the leucovorin group increased from 2.71 to 2.94, while the placebo group decreased from 2.82 to 2.70, consistent with improved methylation potential relative to placebo. SAH decreased in the leucovorin group relative to the placebo (d = −0.67, *p* = 0.050, q = 0.125), from 20.99 to 20.27 nmol/L on leucovorin while increasing from 19.71 to 20.82 nmol/L on placebo. Methionine, SAM and homocysteine showed no significant effect (all *p* ≥ 0.30). Because the pre-specified multiplicity plan applies false-discovery-rate correction within this family, the SAM/SAH and SAH findings are reported as nominal and directionally supportive rather than as confirmed treatment effects. Small within-arm changes in the placebo group may reflect regression to the mean, batch-to-batch assay variability, seasonal or dietary variation in folate intake, and the modest sample size, and thus are not interpreted as treatment effects.

**Figure 5:**
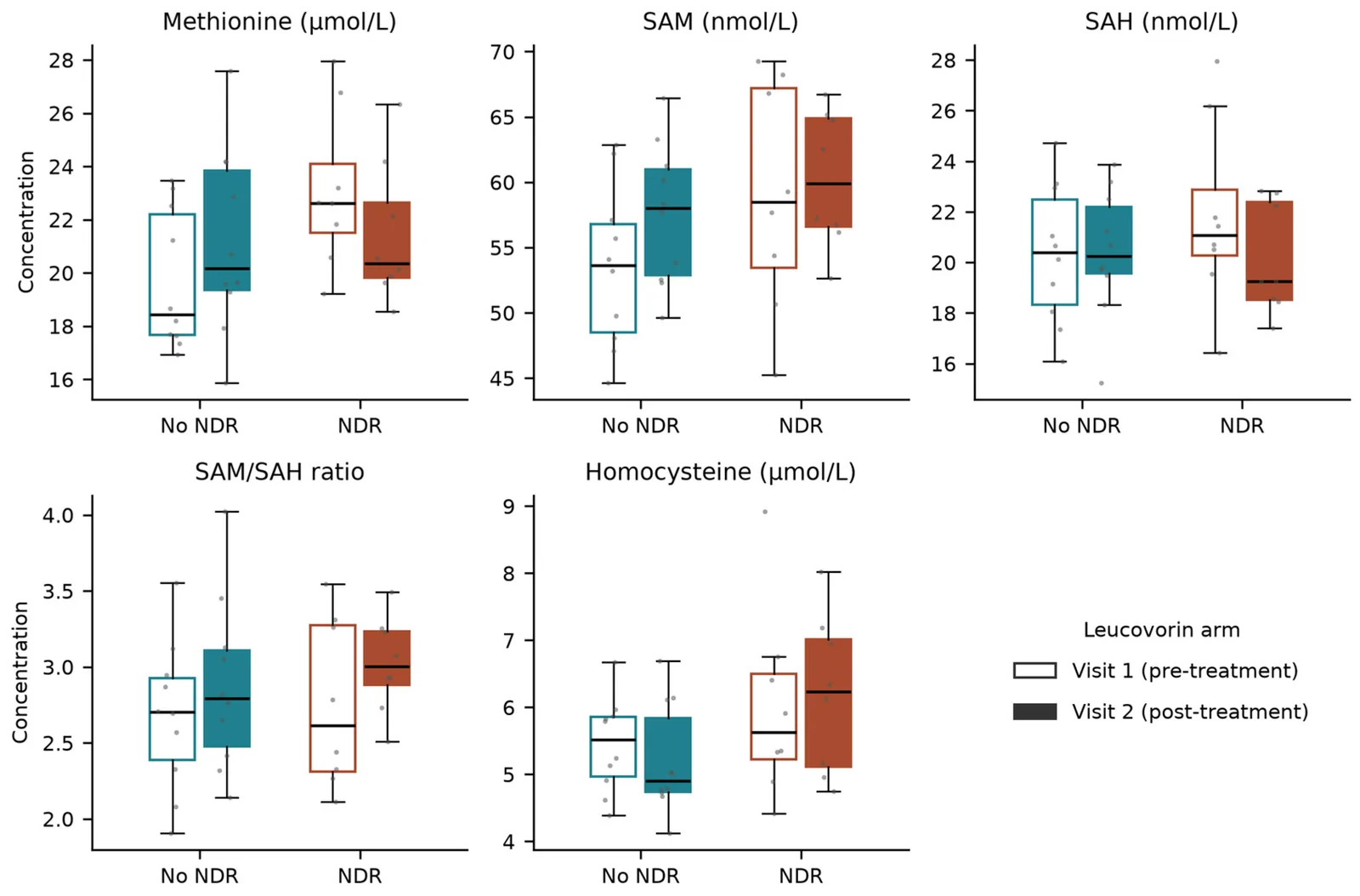
Plasma methylation-cycle metabolites pre- and post-treatment within the leucovorin arm, in children with and without a history of neurodevelopmental regression (NDR). Box plots, shading and individual points are as seen in Figure 4; *n* = 8 with and 10 without a history of NDR. Methionine is the only metabolite whose Group × Visit × NDR interaction survives correction across the thiol family (Supplementary Table S5); the subgroups converge post-treatment.

Within the leucovorin arm, the change in thiol metabolites differed by NDR history (Figure 5; Supplementary Table S5). Methionine was the only metabolite for which the Group × Visit × NDR interaction survived false-discovery-rate correction across the five-member thiol family (β = −5.50, SE 1.75, *p* = 0.002, q = 0.009, d = −1.90): leucovorin increased methionine in children without a history of NDR (19.69 → 21.18 µmol/L, Δ = +1.49) but decreased it in those with a history of NDR (23.10 → 21.42 µmol/L, Δ = −1.68), causing the subgroups to converge post-treatment. The corresponding interactions for SAM, SAH, the SAM/SAH ratio, and homocysteine were not significant (all *p* ≥ 0.21, q ≥ 0.52), so this contrast should be regarded as a single exploratory signal rather than a coherent pattern across the methylation cycle.

### 2.4. Sensitivity and Confirmation Analyses

A complete-case sensitivity analysis restricted to the 26 participants with paired pre- and post-treatment respirometry (11 leucovorin, 15 placebo; 1482 well-level observations) reproduced the primary result: all five respiratory parameters remained significant after correction (BR d = 0.75, ALR d = 0.76, PLR d = 0.46, MRC d = 0.82, RC d = 0.63; all q ≤ 0.023; Supplementary Table S7). Four of the five parameters had q ≤ 0.003 (BR q = 0.0002, ALR q = 0.0001, MRC q = 0.0001, RC q = 0.002); proton leak, the smallest effect in both the primary and the complete-case analysis, had q = 0.023. All 42 participants in the thiol family contributed paired observations, so the thiol analysis is complete set by construction. Sensitivity analyses stratified by baseline concomitant medication use are reported in Supplementary Tables S6 and S6a and in Section 3.4.

### 2.5. Open-Label Extension: Exploratory Within-Participant Analysis

The open-label extension compares a placebo phase (Visit 1 to Visit 2) with an open-label leucovorin phase (Visit 2 to Visit 3) within participants originally randomized to the placebo. Of the 22 participants in that arm with any respirometry, 15 contributed in the placebo phase and 13 in the open-label phase, but only 11 contributed respirometry at all three visits and therefore supplied a complete within-participant phase contrast (NDR yes = 7, NDR no = 4). Using one paired value per participant, no respiratory contrast between the open-label leucovorin phase and the preceding placebo phase was significant (basal Δ = −2.62, ATP-linked Δ = +4.03, proton-leak Δ = −6.65, maximal Δ = −2.51, reserve Δ = +0.11; all *p* ≥ 0.23, all |d| ≤ 0.38), and NDR did not moderate any contrast (all interaction *p* ≥ 0.23). The participant-level paired contrasts reported above, with their standard errors and 95% confidence intervals, are the analysis of record for the extension, and they are null.

In the thiol family, 17 participants originally randomized to placebo contributed plasma at all three visits (NDR yes = 9, NDR no = 8). Across these participants, the open-label leucovorin phase was associated with a larger increase in the SAM/SAH ratio than the preceding placebo phase (Δ = +0.36, d = +0.42, *p* = 0.100) and a larger increase in methionine (Δ = +1.75, d = +0.42, *p* = 0.100), with SAH and homocysteine decreasing non-significantly (Δ = −2.03 and −0.48; *p* = 0.214 and 0.324). The direction of the SAM/SAH contrast matches the randomized comparison, but no contrast reached significance and NDR did not moderate any thiol phase contrast (all interaction *p* ≥ 0.28). Two Visit 3 participant identifiers carried a record-level prefix that prevented them from matching their own earlier visits, which reduced the NDR-negative stratum from eight participants to six; with the identifiers reconciled, no stratum-specific thiol effect is significant (Supplementary Table S8).

## 3. Discussion

This exploratory substudy of a completed DBPC trial found that leucovorin treatment was associated with increases in PBMC mitochondrial respiration and with favorable changes in methylation-cycle metabolites in children with ASD and language impairment. The most consistent respiratory changes were for the energy-producing parameters BR, ALR, MRC and RC. Proton-leak respiration also increased, but by the smallest margin of the five parameters (+4.08, *p* = 0.012, q = 0.012, d = 0.48), so the effect was not confined to the energy-producing parameters. The two arms differed in the linear and quadratic shape of the DMNQ dose-response for MRC and RC, but those are baseline differences between the arms rather than treatment effects; the treatment-related Group × Visit × DMNQ and Group × Visit × DMNQ^2^ terms were null for every parameter. These data therefore provide no evidence that leucovorin altered mitochondrial resilience to in vitro oxidative stress. Given the modest sample size, the exploratory nature of the subgroup analyses and the peripheral tissue examined, these findings should be interpreted as associations that generate hypotheses about the folate–mitochondrial axis rather than as evidence of a specific causal mechanism.

Clinical responders and non-responders differed in the magnitude of their mitochondrial change, but the difference was baseline dependent. Non-responders showed the larger post-treatment increases in MRC and RC while also entering treatment with substantially lower MRC and RC than responders, so the two strata did not start from comparable positions, and the magnitude of their change cannot be compared directly. The verified data do not show that the two subgroups differed in the stability of respiration under DMNQ challenge, and we do not interpret them as doing so. Because responder status is determined after randomization by the clinical outcome itself, this comparison is descriptive; it cannot establish whether mitochondrial changes drive clinical response, are downstream of it, or reflect a shared underlying biology, and it is not offered as mechanistic evidence. The baseline difference between the subgroups is nonetheless worth noting against the mitochondrial phenotypes previously described in subsets of children with ASD and NDR [3,12].

The responder analyses should be read in the context of the parent trial’s broader clinical findings [5]. Responder status here is the parent trial’s own designation—a five-standardized-point increase in verbal communication on the ability-appropriate primary instrument. The parent trial also reported significantly greater improvement on leucovorin than on the placebo for VABS-II Daily Living Skills, several Aberrant Behavior Checklist subscales and total score, Autism Symptom Questionnaire stereotypic behavior and total score, and Behavioral Assessment System for Children internalizing problems, so clinical benefit in the parent trial was not restricted to a single instrument [5]. The mitochondrial and methylation associations reported here are nonetheless anchored to the primary verbal-communication endpoint, and we do not claim that they track global clinical improvement across all domains of ASD.

Within the leucovorin arm, children with a history of NDR improved less than those without such a history for all five respiratory parameters, including reserve capacity. The moderation reached significance for BR, PLR, and RC, but not for ALR (*p* = 0.514) or MRC (*p* = 0.446). For reserve capacity, the interaction is positive in sign only because NDR history was associated with a marked reserve-capacity decline under the placebo, which leucovorin appeared to offset without restoring the gain seen in children without NDR (Supplementary Table S3a). This is consistent with the hypothesis that NDR reflects a distinct mitochondrial pathophysiology characterized by unstable, elevated respiratory rates with vulnerability to metabolic decompensation [12]. Because NDR was classified from the parent report, subject to recall bias and telescoping of timing, and because the NDR subgroups within the leucovorin arm are small, this pattern is offered as an exploratory observation to be tested in larger cohorts with prospective developmental assessment.

### 3.1. Biological Mechanisms Linking Folate to Mitochondrial Function

The observed respiratory changes are broadly consistent with the established role of folate metabolism in mitochondrial function. Folate-dependent OCM (FOCM) is a compartmentalized metabolic network spanning the cytosol, nucleus and mitochondrion, supporting nucleotide biosynthesis, methylation reactions, amino acid interconversion and redox homeostasis [4,13]. Several mechanisms are relevant.

First, mitochondrial FOCM supports mitochondrial DNA (mtDNA) integrity and replication. The mitochondrion has its own folate-dependent thymidylate (dTMP) synthesis pathway that provides nucleotide precursors for mtDNA synthesis and repair [13]. Disruption of this pathway, for example, through deletion of mitochondrial serine hydroxymethyltransferase (SHMT2), leads to mtDNA depletion and uracil misincorporation into mtDNA, compromising mitochondrial genome stability [13]. Folate deprivation reduces mtDNA content and increases large-scale mtDNA deletions across multiple tissues, including the brain [13,14]. Because mtDNA encodes 13 essential ETC subunits, impaired mtDNA integrity can directly compromise oxidative phosphorylation capacity.

Second, mitochondrial FOCM supports the assembly and function of ETC complexes. Mitochondrial FOCM generates N-formylmethionine-tRNA, required for the initiation of translation of mitochondria-encoded proteins, including subunits of complexes I, III, IV and V [15]. SHMT2 deletion reduces steady-state mature complex I, an effect rescued by exogenous formate [13]. In mice, reduced Shmt2 expression lowered mitochondrial folate content and markedly increased uracil in mitochondrial DNA, and in mouse embryonic fibroblasts, reduced Shmt2 expression lowered mitochondrial membrane potential and the oxygen consumption rate [16]. In rats, folate deprivation reduced complex IV activity and caused mitochondrial membrane depolarization, both reversed by folate supplementation [17]. Complex IV is also the site of electron donation by 10-formyl-tetrahydrofolate [18].

Third, folate metabolism contributes to mitochondrial and cellular redox homeostasis. Mitochondrial FOCM, through SHMT2, MTHFD2 and ALDH1L2, generates NADPH used to regenerate reduced glutathione [4,19]. Mitochondrial NADPH production is particularly important under metabolic stress [20]. Folate deficiency suppresses the mitochondrial glutathione pool by impairing biosynthesis and NADPH-dependent recycling [21].

Fourth, FOCM supplies methyl donors for epigenetic regulation [4]. DNA methylation controls the expression of nuclear-encoded mitochondrial genes, including biogenesis regulators such as PGC-1α and TFAM [22]. Disrupted folate status can therefore alter the epigenetic landscape governing mitochondrial biogenesis and turnover [23].

Fifth, folate is integral to mitochondrial stress-response pathways. High-dose folate supplementation suppresses cytochrome c release and caspase-3 activation in endothelial cells challenged with 7-ketocholesterol [24]. In a Drosophila model, folate supplementation and the overexpression of the mitochondrial folate enzyme NMDMC rescued mitochondrial function in neurons and suppressed the integrated stress response [25]. Dietary folic acid supplementation restored mtDNA content and normalized complex IV subunit expression in a porcine model of intrauterine growth restriction [26].

The folate–mitochondria relationship is bidirectional. Mitochondrial dysfunction can impair folate metabolism. The FRα-mediated transport of folate across the blood–brain barrier requires ATP generated by functional mitochondria [5,27]. Mitochondrial disorders such as Kearns–Sayre syndrome and POLG mutations are associated with cerebral folate deficiency due to insufficient energy for active folate transport [27]. Complex I deficiency impairs mitochondrial FOCM by disrupting the NAD^+^/NADH ratio required for MTHFD2 activity, which reduces formate and NADPH production [28] and creates a self-reinforcing cycle.

### 3.2. Thiol Metabolites and One-Carbon Metabolism

The nominal changes in SAH and the SAM/SAH ratio are directionally consistent with the effect of leucovorin on FOCM in children with ASD, but neither survived false-discovery-rate correction within the thiol family and neither should be read as established biochemical target engagement. The increase in the SAM/SAH ratio is consistent with improved methylation potential. SAH is a potent product inhibitor of methyltransferases [4]; the reduction in SAH in the leucovorin group is consistent with improved methylation cycling and reduced inhibitory tone on methylation reactions.

The convergence of the thiol and mitochondrial findings provides a coherent, if hypothesis-generating, mechanistic framework. Leucovorin may improve FOCM, which in turn supports methylation-dependent mitochondrial biogenesis and antioxidant defense through the trans-sulfuration pathway; these metabolic changes may parallel the enhanced mitochondrial respiration observed in the bioenergetic analyses. This account is compatible with, but not directly demonstrated by, the current dataset.

The observation that children with a history of NDR showed less stable mitochondrial physiology and a decrease rather than increase in methionine after leucovorin is on its face paradoxical given the greater supply of folate precursors. One possibility is that the associated increase in mitochondrial respiration in the NDR subgroup increases oxidative demand and shifts flux toward transmethylation and trans-sulfuration, drawing down methionine. This is consistent with our LCL model of ASD in which the high-respiration subgroup showed higher intracellular reactive oxygen species [3]. This mechanistic hypothesis requires direct testing in future work.

### 3.3. Implications for Autism Spectrum Disorder

Folate and mitochondrial abnormalities are individually prevalent in ASD and appear to interact in clinically meaningful ways. Cerebral folate deficiency and compromised FRα function due to folate receptor alpha autoantibodies or mitochondrial dysfunction are relatively common in ASD [27]. Leucovorin is used in the clinical management of mitochondrial disease [29] and has been associated with symptomatic improvement in individuals with ASD and cerebral folate deficiency in observational series.

Inadequate folate may compound existing mitochondrial dysfunction or contribute to its development in predisposed individuals [13,16]. Children with ASD and mitochondrial dysfunction may be particularly vulnerable to folate insufficiency because compromised mitochondria may not generate sufficient ATP for energy-dependent FRα-mediated folate transport, potentially depleting OCM required to sustain mitochondrial function [27].

Direct evidence that folate modulates mitochondrial function in ASD is available. Delhey et al. [30] examined mitochondrial enzyme activities in 127 children with ASD and reported that folate supplementation was associated with higher complex I activity, particularly in the subgroup with confirmed mitochondrial disease, and with differences in the coupling between complex I and citrate synthase [30]. Supplement use in that cohort was observed rather than assigned, so those associations are open to confounding by indication. These findings are consistent with the present observation of increased BR, ALR, MRC and RC with leucovorin treatment, although direct comparison across studies is limited by differences in preparation, tissue and assay.

Children with ASD have been reported to show reduced SAM/SAH ratios, elevated SAH, and depleted glutathione [11,31]. The present findings are consistent with the partial normalization of these methylation-cycle abnormalities during leucovorin treatment. SAM-dependent methylation regulates the expression of neurodevelopmentally relevant genes [22], providing a plausible bridge between biochemical and clinical effects; this bridge remains to be demonstrated directly.

Clinical responders in this substudy may represent a subgroup with more pronounced folate-dependent metabolic dysfunction in whom the restoration of folate status is associated with measurable improvement in both mitochondrial function and communication. The parent trial reported that FRAA-positive children showed the greatest clinical benefit from leucovorin [5]. FRAA status was available in the present substudy, and, consistent with that finding, FRAA-positive children receiving leucovorin showed the largest gains in maximal respiratory capacity and reserve capacity, whereas FRAA-positive children receiving a placebo declined in both (Section 2.2.4). This convergence between the parent trial’s clinical predictor and an independent mitochondrial readout is the most mechanistically informative signal in the dataset, though it rests on small strata and requires replication.

### 3.4. Limitations

This study has several important limitations that must be considered when interpreting the findings, as pre-specified in the exploratory analyses.

First, this is an exploratory biomarker substudy of a single small, randomized trial. Thirty-eight participants contributed mitochondrial respirometry data (17 leucovorin, 21 placebo), of whom 33 contributed at the pre-treatment visit (15 leucovorin, 18 placebo) and 26 contributed paired observations at both visits (11 leucovorin, 15 placebo); 42 participants contributed plasma thiol data (18 leucovorin, 24 placebo). All three respirometry denominators are stated wherever a sample size is reported because the mixed-effects model retains participants with partial data. These samples are well below the large-cohort discovery samples recommended for blood biomarker studies, and the subgroup analyses rest on smaller cell sizes still. Modest *p*-values, particularly in the subgroup analyses, should be interpreted with caution, and we acknowledge that larger samples might not replicate these findings. Effect sizes with 95% confidence intervals are reported throughout to aid interpretation. Randomization also left a material imbalance in baseline respiration, with the placebo arm higher in four of the five parameters (Section 2.2.1). The primary contrast is a difference in within-participant change, which removes the additive effect of that imbalance, but it does not eliminate the possibility that an arm starting further from the cohort mean moves back towards it over the trial interval. Some part of the estimated treatment effect may therefore reflect regression to the mean rather than treatment, and with 17 and 21 participants, the two cannot be separated in these data. This is further reason to regard the magnitude of the effect as distinct from its direction, as provisional.

Second, multiple mitochondrial, methylation and subgroup analyses were performed. Benjamini–Hochberg false discovery rate correction was applied within the primary respiratory and thiol families, and both unadjusted *p*-values and q-values are reported. Among the exploratory analyses, the thiol moderator analyses (Supplementary Table S5) also carry false-discovery-rate correction within the thiol family, whereas the respiratory subgroup and moderator analyses (Supplementary Tables S2, S3 and S9) were not subject to formal multiplicity adjustment. All exploratory analyses are hypothesis-generating only.

Third, the respirometry dataset required source-data verification before analysis. Three defects were identified in the respirometry export: 34 observations recorded at DMNQ concentrations outside the seven-point protocol grid; 121 sets of duplicate wells, involving 254 records and comprising 133 excess copies, in which wells shared a participant, visit, and DMNQ concentration and agreed to six or more decimal places; and one participant visit whose 40 records were numerically identical across all five respiratory outcomes at every DMNQ concentration. Exclusions were applied sequentially to the 1939 randomized-phase records: removing the 34 off-grid records left 1905; removing the 94 duplicates remaining among those left 1811; and removing the six residual records of the degenerate participant visit left the 1805-record analysis set of 38 participants (Section 4.6). Two bases are quoted and should not be conflated. Counted across the full export, including Visit 3, there are 121 duplicate sets involving 254 records, of which 133 are excess copies. Counted within the randomized phase alone, there are 82 duplicate sets involving 176 records, of which 94 are excess copies, and it is these 94 that appear in the sequential cascade above; the 40 degenerate records are likewise a full-export count, of which six survive to the final step. The mean treatment effects are insensitive to these exclusions, whereas the dose-response interaction terms are not, and no differential dose-response is claimed here; the models fitted before and after the exclusions are reproducible from the analysis code, which is available upon reasonable request. Source-data verification also identified an error in the age field of the respirometry export, in which an inadvertent spreadsheet fill had increased age by one year per record for 13 of the 38 participants. Age was therefore taken from the participant-level demographic file of the record, which is single-valued per participant and agrees across two independent exports; no other participant-level variable was affected, and no primary result depends on age. The exploratory age-moderation analysis reported here uses these participant-level ages (Supplementary Table S9, Panel A). Analyses of well-level extracellular-flux data are sensitive to replicate integrity, and we recommend that comparable studies document the provenance of replicate wells.

Fourth, the study does not include a typically developing comparison group and therefore cannot establish whether the folate–mitochondrial relationship described here is specific to ASD. The randomized, placebo-controlled, within-participant design is appropriate for the question actually asked—whether leucovorin changes mitochondrial and one-carbon biochemistry—for which the relevant controls are the concurrent placebo arm and each child’s own pre-treatment measurement. A typically developing arm would be required to address ASD-specificity, but establishing one for the treatment question would entail 12 weeks of high-dose leucovorin in healthy children with no prospect of direct benefit, which we do not consider justifiable in a pediatric population. The complementary case-control question has instead been addressed in dedicated well-matched case-control and sibling-control cohorts using the same DMNQ challenge protocol [12], and the present treatment effects should be read against that literature rather than as evidence of disease specificity.

Fifth, PBMCs are a mixed and dynamic population, and PBMC oxygen consumption can be influenced by immune-cell subset composition and activation state. Immunophenotyping was not performed and cell-mixture effects cannot be excluded. More broadly, PBMC mitochondrial measures may not reflect central nervous system bioenergetics; the discussion of leucovorin effects on brain mitochondria is therefore inferential, and concurrent measurement of central biomarkers such as cerebrospinal fluid folate or neuroimaging correlates of energy metabolism is a priority for future work.

Sixth, the DMNQ oxidative stress model has been validated for characterizing mitochondrial vulnerability in vitro but does not recapitulate the complex in vivo oxidative environment.

Seventh, clinical responder status is the parent trial’s pre-specified verbal-communication responder designation. Although the parent trial also reported significant benefit on several secondary behavioral instruments, the biological correlates described here pertain specifically to change on the primary endpoint and should not be read as correlates of global clinical improvement.

Eighth, NDR was classified from the parent report and is subject to recall bias, telescoping of timing and definitional inconsistency; NDR findings are exploratory and require prospective validation [32].

Ninth, previous work from our group and others indicates that elevated mitochondrial respiration in ASD can reflect compensatory or dysregulated physiology rather than improved function. Interpretation of the observed increases in respiration must accommodate both possibilities, and the divergent responder and non-responder profiles described in Section 2.2.2 illustrate why an increase in respiration should not be read as uniformly favorable.

Tenth, durability cannot be assessed from these data, and the open-label extension is weaker evidence than its model output suggests. The Visit 3 assessment occurred during continued open-label leucovorin exposure rather than after withdrawal, so it cannot establish persistence of effect. Only 11 participants contributed respirometry at all three visits, so the respiratory phase contrast rests on four to seven participants per NDR stratum, and the thiol phase contrast rests on seventeen. Although the assays were performed blind to allocation and phase, treatment in the extension was open-label, and the comparison is confounded by order and by the shared Visit 2 measurement that ends the placebo phase and begins the leucovorin phase. It is reported as directionally consistent with the randomized analysis rather than as confirmation.

Eleventh, the age range is wide (3.4–13.3 years) and spans substantial developmental variation in both mitochondrial physiology and one-carbon metabolism. The study was not designed or powered to characterize age-dependence of the treatment response, and the exploratory age-moderation analysis is reported on that basis.

Twelfth, the sample is heavily male-weighted (34 of 42 participants, 81%; eight girls), reflecting the sex ratio of ASD diagnosis and the referral pattern of the parent trial. With three girls in the leucovorin arm, the study cannot establish whether the biochemical response to leucovorin is comparable in girls. Because ASD is under-recognized in girls and treatment is correspondingly delayed, we regard this as a substantive gap rather than a technical one.

Thirteenth, baseline concomitant medication is reported in Supplementary Table S6 for the nine classes the medication log ascertained. No statistically detectable between-arm differences were found in any recorded class (all Fisher exact *p* ≥ 0.30); with 17 and 21 participants per arm, these comparisons have low power and exclude only large imbalances. Several of the agents recorded are known to influence mitochondrial respiration. Supplement use is not a candidate explanation because vitamin or dietary supplementation exceeding Recommended Dietary Allowance levels was an exclusion criterion of the parent trial [5]. The log does, however, record agent classes rather than individual drugs, so valproate cannot be separated from the other antiepileptic agents. Sensitivity analyses excluding participants receiving psychotropic medication (stimulant, alpha-adrenergic agonist, SSRI or antiepileptic; *n* = 29), additionally excluding melatonin (*n* = 27), and that were restricted to medication-free participants (*n* = 20) all reproduced the primary result with equal or larger effect sizes, and medication use did not moderate the treatment effect (all Group × medication × Visit *p* ≥ 0.097; Supplementary Table S6a). Residual confounding by unrecorded exposures, dose or adherence cannot be excluded.

Finally, the analyses reported here are hypothesis-generating associations. They do not establish that mitochondrial or methylation changes cause clinical improvement, and the causal direction between biology and clinical response cannot be resolved with the current design. FRAA findings are reported in Section 2.2.4 but derive from the strata of seven to seventeen participants. The respiratory FRAA moderation is reported without multiplicity adjustment; the thiol FRAA moderation carries false-discovery-rate correction within the thiol family, where the effect on methionine survives correction (q = 0.012) and the effect on the SAM/SAH ratio does not (q = 0.091). In either case, they should be treated as the basis for a prospective, FRAA-stratified study rather than as established moderation.

## 4. Materials and Methods

### 4.1. Study Design and Participants

This report is a pre-planned exploratory biomarker substudy of a previously reported DBPC trial of leucovorin in children with ASD and language impairment [5]. The parent trial randomized 48 children with ASD to leucovorin (2 mg/kg/day, maximum 50 mg/day; referred to as high-dose folinic acid in Frye et al. [5]) or a matched placebo for 12 weeks, with clinical assessments pre- and post-treatment. This substudy used all biological samples that were successfully collected and processed during the parent trial. Sample availability was constrained by venipuncture failure and insufficient sample volume rather than by any post-randomization selection. A participant flow diagram summarizing the number of participants randomized, sampled and analyzed for each assay is provided as Supplementary Figure S1. Of the 48 randomized children, PBMC samples suitable for Seahorse respirometry were successfully obtained and passed source-data verification for 38 participants at one or both randomized-phase visits (17 leucovorin, 21 placebo), of whom 33 contributed at the pre-treatment visit (15 leucovorin, 18 placebo) and 26 contributed paired observations at both visits (11 leucovorin, 15 placebo), and plasma samples suitable for thiol measurement were obtained from 42 participants (18 leucovorin, 24 placebo). The difference in group sizes reflects differential sample attrition and not the randomization ratio, which was 1:1. All randomized-phase samples were obtained and processed, while treatment allocation was concealed from participants, families and study staff, so this attrition is unlikely to be related to assigned treatment; differential attrition nevertheless remains a potential source of selection bias and is addressed among the limitations. The parent trial had a two-phase design: during Phase 1 (Weeks 0–12), participants were randomized in a double-blind manner to leucovorin (Group A) or a matched placebo (Group B) and were assessed at pre-treatment (Visit 1) and post-treatment (Visit 2). Participants who continued on open-label leucovorin after Phase 1 were reassessed at Visit 3; these Group B Visit 2 → Visit 3 within-participant contrasts constitute the exploratory open-label extension reported in Section 2.5. All respirometry and plasma thiol measurements were performed by laboratory personnel blind to treatment allocation, to study phase, and to clinical outcome, including for the open-label extension. Concomitant medication use upon study entry was ascertained from the parent-trial case report forms, recorded per participant, and grouped into the nine agent classes listed in Supplementary Table S6. The parent trial excluded children taking vitamin or dietary supplementation above Recommended Dietary Allowance levels [5], so no participant was receiving high-dose supplements; these baseline records are the basis of the medication-restricted sensitivity analyses reported in Section 2.4 and Supplementary Table S6a.

Two ClinicalTrials.gov registrations apply to the work reported here. NCT01602016 (A Folinic Acid Intervention for Autism Spectrum Disorders) covers the parent double-blind placebo-controlled trial of leucovorin in children with ASD and language impairment, including the subsequent open-label treatment phase that provided the Visit 3 observations. NCT02000284 (Mitochondrial Dysfunction in Autism Spectrum Disorder) covers the mitochondrial assay protocol under which PBMC respirometry and the associated redox measurements were obtained. Both were approved by the Institutional Review Board of the University of Arkansas for Medical Sciences (Little Rock, AR, USA): the mitochondrial assay protocol (NCT02000284) as protocol 136002 on 19 March 2012, and the parent trial (NCT01602016) as protocol 137162 on 15 August 2012. Written informed consent was obtained from parents or legal guardians, and assent was obtained from children where developmentally appropriate. For the present analysis, all dates were transformed to age in days at visit and all direct personal health identifiers were removed; the resulting de-identified dataset met criteria for exemption 4 under 45 CFR 46.104(d)(4).

#### 4.1.1. Blood Collection and PBMC Isolation

Up to 20 mL of venous blood was collected into EDTA Vacutainer tubes and immediately chilled on ice. Within 30 min of collection, samples were centrifuged at 1500 *g* for 10 min at 4 °C to separate plasma. Plasma was removed, flash frozen and stored at −80 °C. The plasma layer was replaced with room-temperature wash buffer (Ca^2+^/Mg^2+^-free PBS with 0.1% BSA and 2 mM EDTA) and the diluted blood was mixed by gentle inversion.

The diluted blood was layered onto Histopaque-1077 (Sigma-Aldrich, St. Louis, MO, USA) and centrifuged at 400 *g* for 30 min at room temperature. PBMCs were collected from the interface and washed twice with wash buffer. Viable cells were counted using a hemacytometer and trypan blue exclusion. The total isolation procedure required approximately 90–120 min. The Seahorse assay was performed within 4 h of blood collection using EDTA tubes, as previously validated by Werner et al. [33]. PBMC subset composition was not quantified, and cell-mixture effects are addressed in the Limitations section.

#### 4.1.2. Seahorse XFe96 Plate Preparation

PBMCs were resuspended in assay medium consisting of unbuffered RPMI without phenol red; supplemented with 1 mM pyruvate, 2 mM glutamate, and 25 mM glucose; warmed to 37 °C; and pH-adjusted to 7.4. XFe96 plates (Agilent Technologies, Santa Clara, CA, USA) were prepared by adding 25 µL of 50 µg/mL poly-D-lysine (EMD Millipore, Burlington, MA, USA) to each well and incubating for 2 h. Plates were washed with 250 µL sterile water and dried in a laminar flow hood.

Each well was seeded with 4 × 10^5^ viable PBMCs. Plates were spun with slow acceleration (4 on a scale of 9) to a maximum of 100 *g* for 2 min with zero braking, reversed, and spun again in the same fashion. Wells were visualized with an inverted microscope to confirm even cell distribution. At least four replicate wells per condition were seeded. Runs with probe failure, injection failures or non-physiological measurements (ATP-linked respiration or proton leak respiration below −1 pmol/min) were excluded.

#### 4.1.3. Seahorse Test Protocol

The standard Seahorse protocol used a four-step sequential injection. First, baseline oxygen consumption rate (OCR) was measured over three cycles [34,35]. Second, oligomycin (a complex V inhibitor) was injected. Third, carbonyl cyanide-p-trifluoromethoxyphenyl-hydrazone (FCCP) was injected to collapse the mitochondrial inner-membrane potential gradient and measure maximum OCR. Fourth, antimycin A and rotenone were co-injected to shut down the ETC and measure non-mitochondrial OCR.

Five respiratory parameters were derived: (1) basal respiration (BR) = baseline OCR—non-mitochondrial OCR; (2) ATP-linked respiration (ALR) = baseline OCR—OCR after oligomycin; (3) proton leak respiration (PLR) = OCR after oligomycin—non-mitochondrial OCR; (4) maximal respiratory capacity (MRC) = maximal OCR—non-mitochondrial OCR; and (5) reserve capacity (RC) = maximal OCR—baseline OCR.

### 4.2. Mitochondrial Oxidative Stress Test (MOST)

PBMCs were exposed to DMNQ (Sigma-Aldrich, St. Louis, MO, USA) at concentrations of 0, 10, 12.5, 15, 20, 25 and 30 µM. Cells were incubated with DMNQ for 1 h at 37 °C in a non-CO_2_ incubator before the Seahorse assay. A 5 mg/mL DMNQ stock was diluted in DMEM XF assay medium (Agilent Technologies) to create 10× working stocks. DMNQ generates intracellular reactive oxygen species by redox cycling. This approach has been validated for characterizing mitochondrial vulnerability in ASD [12].

### 4.3. Thiol Metabolite Measurement

Plasma metabolites of the methylation cycle—methionine, SAM, SAH, the SAM/SAH ratio and homocysteine—were measured by high-performance liquid chromatography with electrochemical detection, as previously described [11]. Folate receptor alpha autoantibody (FRAA) status was determined in the parent trial for all participants in this substudy and was available for analysis here; blocking and binding autoantibody titers were measured as previously described, and participants were classified as FRAA-positive if either titer exceeded zero [5]. The parent trial reported that FRAA-positive participants showed the greatest clinical benefit from leucovorin [5]; FRAA status is examined as an exploratory moderator of the biomarker outcomes in Section 2.2.4.

### 4.4. Responder Classification

Clinical responder status in this substudy is taken directly from the parent trial [5] and is identical to the responder definition published there; no substudy-specific definition was created. The parent trial’s pre-specified primary outcome was change in verbal communication, measured with an ability-appropriate standardized instrument selected by a structured algorithm—the Clinical Evaluation of Language Fundamentals–Preschool-2, the Clinical Evaluation of Language Fundamentals-4, or the Preschool Language Scale-5—and response was defined a priori as an increase of five-standardized points on that instrument, the threshold the parent trial adopted as a clinically meaningful change. Every participant in the present substudy carries the responder designation assigned in the parent trial under that definition. The parent trial additionally reported significantly greater improvement on leucovorin than on a placebo for VABS-II Daily Living Skills, several Aberrant Behavior Checklist subscales and its total score, Autism Symptom Questionnaire stereotypic behavior and total score, and Behavioral Assessment System for Children internalizing problems [5]. Responder status as used here therefore indexes change on the primary verbal-communication endpoint, while the parent trial’s clinical benefit was not confined to that single domain.

### 4.5. Neurodevelopmental Regression Classification

History of NDR was classified as positive or negative based on parent report of loss of previously acquired skills using the Developmental and Neurobehavioral Regression questionnaire [12]. Parent report of regression is subject to recall bias, telescoping of timing and definitional variability [32]. This limitation is discussed further in the Limitations section, and NDR is treated as an exploratory moderator.

### 4.6. Statistical Analysis

Analyses were organized into an explicit three-level hierarchy. (i) Primary analyses comprise the Group × Visit contrast for the five respiratory parameters (BR, ALR, PLR, MRC, RC) and for the thiol/methylation family; these carry Benjamini–Hochberg false-discovery-rate correction within family and support the inferential claims of the paper. The DMNQ dose-response terms are pre-specified components of the same models and are reported with unadjusted *p*-values: the false-discovery-rate correction is applied to the five Group × Visit contrasts within each outcome family, which is why q-values accompany those five contrasts only. (ii) Sensitivity and confirmation analyses test the robustness of the primary result and comprise the complete-case analysis and the concomitant-medication-restricted analysis. (iii) Exploratory analyses comprise all subgroup and moderator contrasts (clinical responder status, history of NDR, FRAA status, sex and age) together with the single-sequence open-label extension analysis in Group B; these are hypothesis-generating only. They are reported with nominal *p*-values and are not multiplicity-adjusted, with one exception: where a moderator is tested across the five-member thiol/methylation family, Benjamini–Hochberg q-values are reported alongside the nominal *p*-values within that family (Supplementary Tables S5 and S9), and no exploratory claim rests on a nominal *p*-value alone. This hierarchy is signposted in the Results headings, in every table caption, and in every figure caption.

All analyses were conducted in Python 3.14.0 (statsmodels) with restricted maximum likelihood (REML) estimation. The mixed-effects specification used here is the one our group has applied to extracellular-flux respirometry with graded DMNQ challenge in at least ten prior peer-reviewed studies [3], and it is dictated by the structure of the data rather than elective. Observations are well-level oxygen consumption rates nested within DMNQ concentration, within visit, and within participant (at least four replicate wells per condition, seven DMNQ concentrations, two visits); a random participant intercept is the minimum adequate treatment of this nesting, and the intraclass correlations obtained (0.27–0.39; Supplementary Table S1) confirm that between-participant variance is substantial. REML was chosen because sample availability was unbalanced, so that participants with partial observations contribute to fixed-effect estimates rather than being deleted. The quadratic DMNQ term captures the non-monotonic respiratory response to graded oxidative load—a rise at low load followed by a decline as reserve capacity is exhausted—which is a reproducible property of this assay characterized previously [3,12]; the quadratic terms are significant in the fitted models (Supplementary Table S1), confirming that the curvature is estimated rather than assumed. The primary mixed-effects model for respiratory parameters was:

DV ∼ Group × Visit × DMNQ + Group × Visit × DMNQ^2^ + (1|Subject)

where Group (leucovorin, placebo) and Visit (pre-treatment, post-treatment) were categorical, DMNQ and its quadratic term were continuous, and Subject was a random intercept. REML accommodates unbalanced repeated-measure data, so participants with partial observations contribute to fixed-effect estimates rather than being deleted. Thirty-eight participants contributed respirometry at the pre-treatment visit, the post-treatment visit or both (17 leucovorin, 21 placebo); these constitute the primary respirometry analysis sample. Thirty-three contributed at the pre-treatment visit (15 leucovorin, 18 placebo) and 26 contributed paired observations at both visits (11 leucovorin, 15 placebo). The primary model is fitted on all 38 participants; the complete-case sensitivity analysis is restricted to the 26 with paired observations (Supplementary Table S7). All three denominators are stated wherever a respirometry sample size is reported.

Source-data verification. Before analysis, the well-level respirometry export was verified against the assay protocol. Thirty-four records carried DMNQ concentrations not used in the protocol; all belonged to a single participant visit in which all 40 records were numerically identical across all five respiratory outcomes and showed no decline with increasing DMNQ, and that participant visit was therefore excluded in full (40 records). Across the full export, a further 254 records fell into 121 sets in which two or more wells shared the same participant, visit, and DMNQ concentration and agreed to six or more decimal places for all five respiratory outcomes; one member of each set was retained and the remaining 133 excess copies were treated as spreadsheet artifacts rather than independent replicates. Restricted to the randomized phase (Visits 1 and 2), which supplies the primary analysis set, the same rule identifies 82 duplicate sets involving 176 records, of which 94 are excess copies. The analyzed dataset comprises 2290 well-level records, of which 1805 fall in the pre- and post-treatment visits used for the primary model. Two participant identifiers in the thiol export differed from their counterparts only by trailing whitespace; reconciling them increases the number of participants with paired thiol observations from 40 to 42. All estimates reported here derive from this verified dataset.

For thiol metabolites, which involved single measurements per visit, the mixed-effects model was:

DV ∼ Group × Visit + (1|Subject).

Extended models added clinical responder status, history of NDR, FRAA status, sex, age (mean-centered), and their interactions with Group and Visit as exploratory moderators. These models are saturated in the moderator, so a coefficient for the moderator depends on which treatment arm is the reference level. Moderator effects are therefore reported as simple effects within each arm, with the arm stated explicitly, so that every contrast described in the text is drawn from the arm to which it refers (Supplementary Tables S2, S3 and S9).

The association between NDR and clinical responder status was examined with Fisher’s exact tests overall and stratified by treatment group, the Cochran–Mantel–Haenszel test was used to control for the treatment group, and logistic regression with responder status was used for the outcome and groups, with NDR and their interaction as predictors.

Effect sizes are reported as Cohen’s d, computed from the fixed-effect coefficient divided by the total standard deviation (the square root of the sum of random-effect and residual variances), with 95% confidence intervals. To address multiple testing across the primary respiratory (BR, ALR, PLR, MRC, RC) and thiol (methionine, SAM, SAH, SAM/SAH ratio, homocysteine) families, Benjamini–Hochberg false discovery rate correction was applied within each family separately at a within-family false-discovery-rate threshold of q < 0.05. Both unadjusted *p*-values and Benjamini–Hochberg q-values are reported in the Supplementary Tables. Given the modest sample size and the number of subgroup contrasts examined, all subgroup findings (responder and NDR analyses) are interpreted as exploratory and hypothesis-generating rather than confirmatory. Nominal *p*-values in the exploratory subgroup analyses are reported without adjustment and interpreted cautiously as pre-specified exploratory analyses. Within the thiol family, the nominally significant effects on the SAM/SAH ratio and S-adenosylhomocysteine did not survive Benjamini–Hochberg correction (q = 0.098 and q = 0.125) and are described as nominal throughout.

Sensitivity and exploratory extension analyses. To examine the primary Group × Visit findings within participants, we analyzed the open-label extension in Group B. Group B participants received a double-blind placebo during Phase 1 (V1→V2) and open-label leucovorin during Phase 2 (V2→V3), so each contributed a paired contrast of a placebo phase and a subsequent leucovorin phase and served as their own control, eliminating between-participant variance. We describe this as a single-sequence within-participant phase comparison rather than a crossover: no participant received the reverse sequence, and the leucovorin phase was always second and was administered open-label, so period, time and treatment effects are not separable. The laboratory assays themselves were performed blind to allocation and phase, so the limitation is one of design and order rather than of measurement. The analysis is accordingly exploratory and cannot confirm the randomized comparison. Because regression (NDR history) was a pre-specified subject-level moderator in the primary analyses, we retained it as a stratifying factor in the extension analysis. For the thiol/methylation family, we tested the paired-delta contrast Δ_leucovorin (V3 − V2) minus Δ_placebo (V2 − V1) with a paired *t*-test overall and within each NDR stratum and tested the regression × phase interaction with an OLS on the paired-delta difference (95% CIs and Cohen’s d reported). For the respiratory family, we used one paired value per participant, formed as the difference between the leucovorin-phase change (V3 − V2) and the placebo-phase change (V2 − V1) in the participant’s mean respiration across the DMNQ series at that visit, and tested it with a paired *t*-test overall and, by two-sample *t*-test, between NDR strata; standard errors, t-based 95% confidence intervals and Cohen’s d are reported. A well-level mixed-effects model of the same data was examined during analysis but is not reported: it treats wells within a participant visit as independent and reuses each Visit 2 measurement in both phases, so its standard errors are anticonservative, and its *p*-values are not valid inference. Group A received leucovorin during both phases and does not contribute a phase contrast; V2→V3 change in Group A is reported descriptively as a treatment-continuation reference in Supplementary Table S8, Panel C, where only three Group A participants contributed respirometry at both Visit 2 and Visit 3, so that panel is descriptive only and supports no inference. In addition, two further sensitivity analyses were performed for the primary V1→V2 models: a complete-case analysis restricted to participants with paired pre- and post-treatment observations and an analysis restricted to participants not receiving any mitochondrially active or antioxidant agent at baseline (Supplementary Tables S6 and S7).

## 5. Conclusions

In this exploratory substudy of a completed DBPC trial, leucovorin was associated with increases in all five measured parameters of PBMC mitochondrial respiration in children with ASD and language impairment, and with a nominal increase in the plasma SAM/SAH ratio and decrease in S-adenosylhomocysteine that did not survive false-discovery-rate correction. The respiratory effect was robust to complete-case and medication-restricted sensitivity analyses, and there was no evidence that it varied across the tested DMNQ range. Folate receptor alpha autoantibody status and a history of neurodevelopmental regression modified the respiratory response, and clinical responders differed from non-responders in baseline maximal and reserve capacity. These are hypothesis-generating associations rather than confirmatory mechanistic evidence. Two design features would materially strengthen the next study. A randomized withdrawal or delayed-start comparison would separate a continuing treatment effect from period and time effects in a way the single-sequence open-label extension reported here cannot. Prospective enrollment stratified by sex would allow for the biochemical response in girls to be estimated rather than inferred from three participants. Replication in larger, independent, FRAA-stratified cohorts that integrate central nervous system measures and prospective developmental assessment is needed before clinical translation.

## Figures and Tables

**Table 1:** Participant demographics and clinical characteristics at baseline for the biomarker substudy, by treatment arm.

Characteristic	Leucovorin (*n* = 18)	Placebo (*n* = 24)	Total (*N* = 42)
Male, *n* (%)	15 (83%)	19 (79%)	34 (81%)
Female, *n* (%)	3 (17%)	5 (21%)	8 (19%)
Age, mean (SD)	7.6 (3.2)	7.2 (3.0)	7.4 (3.0)
Age range	3.5–13.3	3.4–12.6	3.4–13.3
Responders, *n* (%)	11 (61%)	6 (25%)	17 (40%)
Neurodevelopmental regression, *n* (%)	8 (44%)	12 (50%)	20 (48%)
FRAA positive (blocking or binding), *n* (%)	9 (50%)	17 (71%)	26 (62%)
FRAA blocking > 0, *n* (%)	3 (17%)	4 (17%)	7 (17%)
FRAA binding > 0, *n* (%)	8 (44%)	14 (58%)	22 (52%)

**Table 2:** Primary analysis: effect of leucovorin versus placebo on mitochondrial respiratory parameters in peripheral blood mononuclear cells. Estimates are the Group × Visit interaction from the pre-specified mixed-effects model DV ~ Group × Visit × DMNQ + Group × Visit × DMNQ^2^ + (1|Subject), evaluated at DMNQ = 0 and expressed as the leucovorin-minus-placebo difference in pre-to-post change; positive values denote a greater increase on leucovorin. Cohen’s d = β/√(σ^2^_subject + σ^2^_residual); each effect size is derived line-by-line from the model variance components in Supplementary Table S1a. q-values are Benjamini–Hochberg false-discovery-rate-adjusted within the five-outcome respiratory family. This table reports the primary pre-specified contrasts only; DMNQ dose-response terms, full model output and all exploratory subgroup analyses are given in the Supplementary Tables.

Outcome	Effect (95% CI)	*p*	q (BH)	Cohen’s d (95% CI)	Leucovorin Effect
Basal respiration (BR)	+16.66 (+9.45 to +23.87)	<0.001	<0.001	0.81 (0.46–1.17)	Increased
ATP-linked respiration (ALR)	+12.62 (+7.47 to +17.77)	<0.001	<0.001	0.84 (0.49–1.18)	Increased
Proton-leak respiration (PLR)	+4.08 (+0.90 to +7.26)	0.012	0.012	0.48 (0.11–0.85)	Increased
Maximal respiratory capacity (MRC)	+42.81 (+26.80 to +58.82)	<0.001	<0.001	0.92 (0.57–1.26)	Increased
Reserve capacity (RC)	+25.80 (+12.58 to +39.01)	<0.001	0.0002	0.71 (0.35–1.08)	Increased

## Data Availability

De-identified individual-level data supporting the analysis will be made available upon reasonable request to the corresponding author. The complete statistical analysis code and the derived analytic datasets required to reproduce every result, table and figure reported here, together with a data dictionary and a self-verifying reproduction script, are available from the corresponding author.

## Supplementary Materials

The following supporting information can be downloaded at: https://www.mdpi.com/article/10.3390/ijms27209101/s1.

## Abbreviations

ALR, ATP-linked respiration; ASD, autism spectrum disorder; BR, basal respiration; DBPC, double-blind placebo-controlled; DMNQ, 2,3-dimethoxy-1,4-naphthoquinone; ETC, electron transport chain; FDR, false discovery rate; FOCM, folate-dependent one-carbon metabolism; FRAA, folate receptor alpha autoantibody; FRα, folate receptor alpha; LCL, lymphoblastoid cell line; MOST, mitochondrial oxidative stress test; MRC, maximal respiratory capacity; mtDNA, mitochondrial DNA; NDR, neurodevelopmental regression; OCM, one-carbon metabolism; OCR, oxygen consumption rate; PBMC, peripheral blood mononuclear cell; PLR, proton leak respiration; RC, reserve capacity; REML, restricted maximum likelihood; SAH, S-adenosylhomocysteine; SAM, S-adenosylmethionine; VABS-II, Vineland Adaptive Behavior Scales, Second Edition.
