# Supplementary material for: Effect of Leucovorin on Mitochondrial Respiration and One-Carbon Metabolism in Autism Spectrum Disorder: A Double-Blind Placebo-Controlled Substudy
Source: Int J Mol Sci. 2026 Oct 14;27(20):9101. doi: 10.3390/ijms27209101 (PMC13705312; doi:10.3390/ijms27209101)
Supplement: Supplementary file 1 [file ijms-27-09101-s001.zip › Supplementary-Figure-S1_CONSORT.pdf]

Supplementary Figure S1

Participant flow for the biomarker substudy (CONSORT-style)

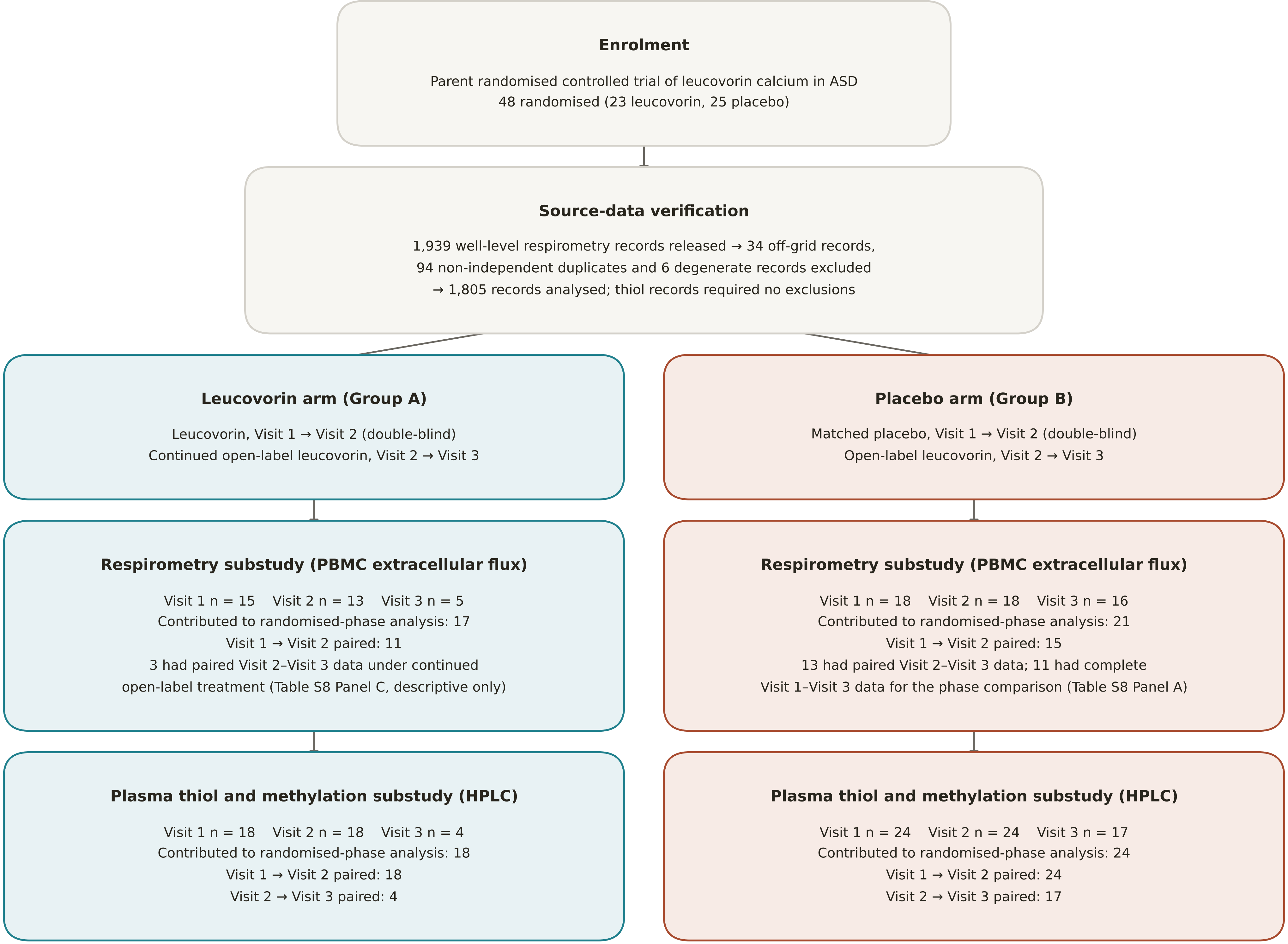

Notes. Visit 1 = pre-treatment baseline; Visit 2 = end of the double-blind phase; Visit 3 = end of the open-label phase, in which all participants received leucovorin. Losses reflect declined or unsuccessful venipuncture or insufficient sample volume for the respective assay.

The primary analysis is a mixed-effects model fitted to all available observations from the 38 participants contributing respirometry (17 leucovorin, 21 placebo) and all 42 contributing thiol measurements (18 leucovorin, 24 placebo); it does not require paired data. The paired counts above define the complete-case sensitivity analysis (26 participants for respirometry; Supplementary Table S7).

The Group B Visit 2 → Visit 3 change is an exploratory open-label comparison, not a confirmation of the double-blind result, and is reported in Supplementary Table S8.

All counts derive from the verified analysis sets generated by the archived analysis code.
