# Supplementary material for: Effect of Leucovorin on Mitochondrial Respiration and One-Carbon Metabolism in Autism Spectrum Disorder: A Double-Blind Placebo-Controlled Substudy
Source: Int J Mol Sci. 2026 Oct 14;27(20):9101. doi: 10.3390/ijms27209101 (PMC13705312; doi:10.3390/ijms27209101)
Supplement: Supplementary file 1 [file ijms-27-09101-s001.zip › Supplementary-Tables-S1-S9_Revised.pdf]

Supplementary Tables

Supplementary Table S1. Overall Mixed-Effects Model Results for Mitochondrial Respiratory Parameters

Note: * p < 0.05, ** p < 0.01, *** p < 0.001. Reference levels are leucovorin and Visit 1 (pre-treatment), so the Group × Visit coefficient is the placebo-minus-leucovorin difference in pre-to-post change; the treatment effect reported in the main text is its negation. Models were fitted by restricted maximum likelihood with a participant random intercept on 1,805 well-level observations from 38 participants after source-data verification. Intraclass correlations were 0.27–0.39.

Parameter / Term	β	SE	p-value
**Basal respiration (BR)**			
Intercept (leucovorin, Visit 1, DMNQ 0)	+31.964	3.463	<0.001***
Group (placebo vs leucovorin)	+12.796	4.717	0.007**
Visit (post vs pre)	+11.309	2.592	<0.001***
Group × Visit	−16.659	3.679	<0.001***
DMNQ	+0.974	0.260	<0.001***
Group × DMNQ	+0.436	0.360	0.226
Visit × DMNQ	+0.264	0.366	0.472
Group × Visit × DMNQ	+0.096	0.512	0.851
DMNQ²	−0.031	0.009	<0.001***
Group × DMNQ²	−0.006	0.012	0.638
Visit × DMNQ²	−0.006	0.012	0.601
Group × Visit × DMNQ²	−0.008	0.017	0.623
**ATP-linked respiration (ALR)**			
Intercept (leucovorin, Visit 1, DMNQ 0)	+29.313	2.641	<0.001***
Group (placebo vs leucovorin)	+11.070	3.592	0.002**
Visit (post vs pre)	+8.317	1.851	<0.001***
Group × Visit	−12.616	2.628	<0.001***
DMNQ	+0.028	0.185	0.879
Group × DMNQ	+0.144	0.257	0.577
Visit × DMNQ	+0.254	0.262	0.332
Group × Visit × DMNQ	−0.088	0.366	0.811
DMNQ²	−0.011	0.006	0.072
Group × DMNQ²	−0.001	0.009	0.870
Visit × DMNQ²	−0.011	0.009	0.209
Group × Visit × DMNQ²	+0.002	0.012	0.872
**Proton-leak respiration (PLR)**			
Intercept (leucovorin, Visit 1, DMNQ 0)	+2.652	1.349	0.049*
Group (placebo vs leucovorin)	+1.749	1.844	0.343
Visit (post vs pre)	+2.988	1.143	0.009**
Group × Visit	−4.076	1.622	0.012*
DMNQ	+0.945	0.115	<0.001***
Group × DMNQ	+0.292	0.159	0.067
Visit × DMNQ	+0.010	0.162	0.949
Group × Visit × DMNQ	+0.183	0.226	0.417
DMNQ²	−0.020	0.004	<0.001***
Group × DMNQ²	−0.004	0.005	0.429
Visit × DMNQ²	+0.004	0.005	0.399
Group × Visit × DMNQ²	−0.010	0.007	0.170
**Maximal respiratory capacity (MRC)**			
Intercept (leucovorin, Visit 1, DMNQ 0)	+101.853	8.164	<0.001***
Group (placebo vs leucovorin)	+58.927	11.106	<0.001***
Visit (post vs pre)	+31.669	5.754	<0.001***

Parameter / Term	β	SE	p-value
Group × Visit	−42.808	8.169	<0.001***
DMNQ	−3.927	0.576	<0.001***
Group × DMNQ	−3.323	0.800	<0.001***
Visit × DMNQ	−0.429	0.813	0.597
Group × Visit × DMNQ	+1.202	1.137	0.290
$DMNQ^2$	+0.050	0.019	0.008**
Group × $DMNQ^2$	+0.082	0.026	0.002**
Visit × $DMNQ^2$	+0.002	0.027	0.955
Group × Visit × $DMNQ^2$	−0.036	0.037	0.333
**Reserve capacity (RC)**			
Intercept (leucovorin, Visit 1, DMNQ 0)	+69.935	5.859	<0.001***
Group (placebo vs leucovorin)	+46.081	7.998	<0.001***
Visit (post vs pre)	+20.255	4.749	<0.001***
Group × Visit	−25.797	6.742	<0.001***
DMNQ	−4.902	0.476	<0.001***
Group × DMNQ	−3.754	0.661	<0.001***
Visit × DMNQ	−0.694	0.672	0.301
Group × Visit × DMNQ	+1.099	0.939	0.242
$DMNQ^2$	+0.081	0.016	<0.001***
Group × $DMNQ^2$	+0.087	0.022	<0.001***
Visit × $DMNQ^2$	+0.008	0.022	0.723
Group × Visit × $DMNQ^2$	−0.027	0.031	0.372

Note on Cohen's d for Supplementary Tables S1–S3 (respiratory outcomes). Cohen's d values reported in the main-text Results and in main-text Table 2 for basal respiration (BR), ATP-linked respiration (ALR), proton-leak respiration (PLR), maximal respiratory capacity (MRC) and reserve capacity (RC) are derived from the Group × Visit interaction β divided by the square root of the sum of the random-intercept and residual variance components of the corresponding mixed-effects model, i.e. $d = \beta / \sqrt{\sigma^2\_subject + \sigma^2\_residual}$. Confidence intervals for d are obtained by applying the same denominator to the 95% confidence interval of β. This convention pools between- and within-subject variability and is the same denominator used for Cohen's d in Supplementary Table S4 (thiols) and Supplementary Table S7 (complete-case sensitivity analysis). Each main-text effect size is derived directly from the β and variance components printed in Supplementary Table S1, so that every reported d is reproducible from this supplement; the derivation is set out line by line in Supplementary Table S1a.

Supplementary Table S1a. Derivation of the main-text effect sizes from the mixed-effects output in Supplementary Table S1. Each d is recomputed as $\beta / \sqrt{\sigma^2\_subject + \sigma^2\_residual}$ using the β, σ²_subject and σ²_residual printed in Supplementary Table S1 for the Group B (placebo) × Visit 2 interaction; the sign is reversed for presentation so that positive d denotes a greater increase on leucovorin.

Outcome	β (Group × Visit)	σ²_subject	σ²_residual	√(σ²_s+σ²_r)	Cohen's d	95% CI for d	ICC
Basal respiration (BR)	+16.66	147.0	271.2	20.45	+0.81	[+0.46, +1.17]	0.352
ATP-linked respiration (ALR)	+12.62	89.6	138.2	15.09	+0.84	[+0.49, +1.18]	0.393
Proton-leak respiration (PLR)	+4.08	19.9	52.8	8.53	+0.48	[+0.11, +0.85]	0.273
Maximal respiratory capacity (MRC)	+42.81	852.9	1335.5	46.78	+0.92	[+0.57, +1.26]	0.390
Reserve capacity (RC)	+25.80	392.6	911.3	36.11	+0.71	[+0.35, +1.08]	0.301

Supplementary Table S2. Responder analysis with DMNQ dose-response, fitted across both arms.

Note: * $p < 0.05$, ** $p < 0.01$, *** $p < 0.001$. Reference levels are placebo, non-responder and Visit 1, so every coefficient is relative to placebo non-responders at baseline. The term Responder × Visit therefore describes responders in the PLACEBO arm, and the corresponding leucovorin-arm contrast is Group × Responder × Visit added to Responder × Visit. Two arithmetically distinct quantities are reported for the same contrast and both are correct. Adding the coefficients printed in this table gives the leucovorin-arm contrast implied by the full four-way model: for maximal respiratory capacity, Responder × Visit (+148.034) + Group × Responder × Visit (−210.826) = −62.79, and for reserve capacity +121.350 + (−178.115) = −56.77; these are the values quoted in the main text. Refitting the model separately within each arm, which uses only that arm's data to estimate its own variance components, gives Responder × Visit =

−62.28 for maximal respiratory capacity and −56.24 for reserve capacity in the leucovorin arm, and +148.64 and +121.67 respectively in the placebo arm (all p < 0.001). The small differences between the two routes reflect the different variance structures, not different data. Responder status is the parent trial designation and these contrasts are exploratory and unadjusted for multiplicity. Fitted on 38 participants; leucovorin 10 responders and 7 non-responders, placebo 5 responders and 16 non-responders.

Parameter / Term	β	SE	p-value
**Basal respiration (BR)**			
Intercept (placebo, non-responder, Visit 1, DMNQ 0)	+48.211	3.785	<0.001***
Group (leucovorin vs placebo)	−14.378	6.578	0.029*
Responder (vs non-responder)	−15.159	7.463	0.042*
Visit (post vs pre)	−8.992	2.831	0.001**
Group × Responder	+12.954	10.314	0.209
Group × Visit	+22.979	4.804	<0.001***
Responder × Visit (placebo arm)	+26.050	8.278	0.002**
Group × Responder × Visit	−31.800	9.741	0.001**
**ATP-linked respiration (ALR)**			
Intercept (placebo, non-responder, Visit 1, DMNQ 0)	+43.331	2.860	<0.001***
Group (leucovorin vs placebo)	−13.090	4.996	0.009**
Responder (vs non-responder)	−12.038	5.667	0.034*
Visit (post vs pre)	−8.012	2.010	<0.001***
Group × Responder	+11.340	7.835	0.148
Group × Visit	+19.746	3.409	<0.001***
Responder × Visit (placebo arm)	+23.963	5.877	<0.001***
Group × Responder × Visit	−30.705	6.915	<0.001***
**Proton-leak respiration (PLR)**			
Intercept (placebo, non-responder, Visit 1, DMNQ 0)	+4.905	1.483	<0.001***
Group (leucovorin vs placebo)	−1.304	2.543	0.608
Responder (vs non-responder)	−3.084	2.885	0.285
Visit (post vs pre)	−1.032	1.272	0.418
Group × Responder	+1.570	3.983	0.693
Group × Visit	+3.287	2.159	0.128
Responder × Visit (placebo arm)	+2.088	3.713	0.574
Group × Responder × Visit	−1.115	4.371	0.799
**Maximal respiratory capacity (MRC)**			
Intercept (placebo, non-responder, Visit 1, DMNQ 0)	+181.259	8.772	<0.001***
Group (leucovorin vs placebo)	−91.614	15.324	<0.001***
Responder (vs non-responder)	−74.864	17.382	<0.001***
Visit (post vs pre)	−33.882	6.165	<0.001***
Group × Responder	+101.249	24.030	<0.001***
Group × Visit	+99.762	10.460	<0.001***
Responder × Visit (placebo arm)	+148.034	18.030	<0.001***
Group × Responder × Visit	−210.826	21.216	<0.001***
**Reserve capacity (RC)**			
Intercept (placebo, non-responder, Visit 1, DMNQ 0)	+133.148	6.354	<0.001***
Group (leucovorin vs placebo)	−77.311	10.966	<0.001***
Responder (vs non-responder)	−59.510	12.439	<0.001***
Visit (post vs pre)	−24.811	5.132	<0.001***
Group × Responder	+88.033	17.183	<0.001***
Group × Visit	+76.510	8.709	<0.001***
Responder × Visit (placebo arm)	+121.350	14.989	<0.001***
Group × Responder × Visit	−178.115	17.644	<0.001***

Supplementary Table S3. Moderation Effect of Neurodevelopmental Regression on Mitochondrial Respiration

Parameter / Term	β	SE	p-value
**Basal respiration (BR)**			
Intercept (placebo, no NDR, Visit 1, DMNQ 0)	+42.453	4.274	<0.001***
Group (leucovorin vs placebo)	−13.411	6.174	0.030*
Visit (post vs pre)	−1.243	2.820	0.659
NDR (vs no NDR)	+5.144	6.074	0.397
Group × Visit	+22.572	4.035	<0.001***
NDR × Visit	−8.210	2.457	<0.001***
Group × NDR	+1.018	9.033	0.910
Group × NDR × Visit	−10.985	3.599	0.002**
**ATP-linked respiration (ALR)**			
Intercept (placebo, no NDR, Visit 1, DMNQ 0)	+38.301	3.290	<0.001***
Group (leucovorin vs placebo)	−10.397	4.755	0.029*
Visit (post vs pre)	+1.334	1.994	0.503
NDR (vs no NDR)	+5.254	4.690	0.263
Group × Visit	+14.369	2.853	<0.001***
NDR × Visit	−12.378	1.738	<0.001***
Group × NDR	−2.284	6.991	0.744
Group × NDR × Visit	−1.662	2.546	0.514
**Proton-leak respiration (PLR)**			
Intercept (placebo, no NDR, Visit 1, DMNQ 0)	+4.167	1.624	0.010*
Group (leucovorin vs placebo)	−3.034	2.344	0.196
Visit (post vs pre)	−2.583	1.247	0.038*
NDR (vs no NDR)	−0.075	2.292	0.974
Group × Visit	+8.220	1.783	<0.001***
NDR × Visit	+4.062	1.087	<0.001***
Group × NDR	+3.286	3.392	0.333
Group × NDR × Visit	−9.254	1.589	<0.001***
**Maximal respiratory capacity (MRC)**			
Intercept (placebo, no NDR, Visit 1, DMNQ 0)	+141.656	10.028	<0.001***
Group (leucovorin vs placebo)	−45.720	14.490	0.002**
Visit (post vs pre)	+5.991	6.196	0.334
NDR (vs no NDR)	+44.709	14.286	0.002**
Group × Visit	+44.350	8.867	<0.001***
NDR × Visit	−42.735	5.401	<0.001***
Group × NDR	−31.933	21.284	0.134
Group × NDR × Visit	+6.030	7.913	0.446
**Reserve capacity (RC)**			
Intercept (placebo, no NDR, Visit 1, DMNQ 0)	+99.200	7.193	<0.001***
Group (leucovorin vs placebo)	−32.222	10.386	0.002**
Visit (post vs pre)	+7.508	5.137	0.144
NDR (vs no NDR)	+39.633	10.188	<0.001***
Group × Visit	+21.249	7.351	0.004**
NDR × Visit	−34.654	4.468	<0.001***
Group × NDR	−33.143	15.116	0.028*
Group × NDR × Visit	+17.444	6.547	0.008**

Note: * $p < 0.05$, ** $p < 0.01$, *** $p < 0.001$. Reference levels are placebo, no neurodevelopmental regression (NDR) and Visit 1. Dose terms (DMNQ, DMNQ^2 and their interactions) were included in the model and are omitted from the table for legibility. NDR was classified from parent report; these contrasts are exploratory and unadjusted for multiplicity.

Supplementary Table S3a. Arm-specific simple effects of the Supplementary Table S3 model: within-arm pre-to-post change by neurodevelopmental regression stratum, and the within-arm NDR contrast.

Note. Every quantity is a linear contrast of the coefficients of the same model reported in Supplementary Table S3, evaluated at DMNQ = 0; the model specification is unchanged. The first two rows of each block give the Visit 1 to Visit 2 change within one treatment arm and NDR stratum, and the third gives the difference between those strata within that arm. The Group × NDR × Visit coefficient of Table S3 equals the leucovorin-arm NDR contrast minus the placebo-arm NDR contrast, so it tests whether NDR-related moderation differs between arms and does not by itself establish the direction or size of change within either arm. Confidence intervals are Wald intervals; Cohen's d is the contrast divided by the total standard deviation. These subgroups are small (7 to 11 participants per cell) and NDR was classified from parent report, so all rows are exploratory. * $p < 0.05$, ** $p < 0.01$, *** $p < 0.001$.

Arm / NDR stratum	n	β	SE	95% CI	p-value	Cohen's d
**Basal respiration (BR)**						
Placebo, no NDR history	11	−1.243	2.820	−6.77 to +4.28	0.659	−0.061
Placebo, NDR history	10	−9.453	2.917	−15.17 to −3.74	0.001**	−0.466
Placebo, NDR minus no NDR	21	−8.210	2.457	−13.03 to −3.39	<0.001***	−0.404
Leucovorin, no NDR history	10	+21.328	2.884	+15.68 to +26.98	<0.001***	+1.050
Leucovorin, NDR history	7	+2.133	2.850	−3.45 to +7.72	0.454	+0.105
Leucovorin, NDR minus no NDR	17	−19.196	2.628	−24.35 to −14.05	<0.001***	−0.945
**ATP-linked respiration (ALR)**						
Placebo, no NDR history	11	+1.334	1.994	−2.57 to +5.24	0.503	+0.089
Placebo, NDR history	10	−11.043	2.063	−15.09 to −7.00	<0.001***	−0.737
Placebo, NDR minus no NDR	21	−12.378	1.738	−15.78 to −8.97	<0.001***	−0.826
Leucovorin, no NDR history	10	+15.704	2.039	+11.71 to +19.70	<0.001***	+1.048
Leucovorin, NDR history	7	+1.664	2.015	−2.29 to +5.61	0.409	+0.111
Leucovorin, NDR minus no NDR	17	−14.040	1.860	−17.69 to −10.39	<0.001***	−0.937
**Proton-leak respiration (PLR)**						
Placebo, no NDR history	11	−2.583	1.247	−5.03 to −0.14	0.038*	−0.308
Placebo, NDR history	10	+1.479	1.292	−1.05 to +4.01	0.252	+0.176
Placebo, NDR minus no NDR	21	+4.062	1.087	+1.93 to +6.19	<0.001***	+0.484
Leucovorin, no NDR history	10	+5.637	1.275	+3.14 to +8.14	<0.001***	+0.671
Leucovorin, NDR history	7	+0.445	1.261	−2.03 to +2.92	0.724	+0.053
Leucovorin, NDR minus no NDR	17	−5.192	1.158	−7.46 to −2.92	<0.001***	−0.618
**Maximal respiratory capacity (MRC)**						
Placebo, no NDR history	11	+5.991	6.196	−6.15 to +18.14	0.334	+0.130
Placebo, NDR history	10	−36.744	6.410	−49.31 to −24.18	<0.001***	−0.797
Placebo, NDR minus no NDR	21	−42.735	5.401	−53.32 to −32.15	<0.001***	−0.927
Leucovorin, no NDR history	10	+50.341	6.337	+37.92 to +62.76	<0.001***	+1.092
Leucovorin, NDR history	7	+13.636	6.261	+1.36 to +25.91	0.029*	+0.296
Leucovorin, NDR minus no NDR	17	−36.705	5.779	−48.03 to −25.38	<0.001***	−0.796
**Reserve capacity (RC)**						
Placebo, no NDR history	11	+7.508	5.137	−2.56 to +17.58	0.144	+0.211
Placebo, NDR history	10	−27.146	5.314	−37.56 to −16.73	<0.001***	−0.761
Placebo, NDR minus no NDR	21	−34.654	4.468	−43.41 to −25.90	<0.001***	−0.972
Leucovorin, no NDR history	10	+28.757	5.252	+18.46 to +39.05	<0.001***	+0.806
Leucovorin, NDR history	7	+11.548	5.193	+1.37 to +21.73	0.026*	+0.324
Leucovorin, NDR minus no NDR	17	−17.209	4.782	−26.58 to −7.84	<0.001***	−0.483

Supplementary Table S4. Thiol Metabolite Mixed-Effects Model Results: Group × Visit Interaction

Note. The Group × Visit interaction (β) is the differential Visit 1 to Visit 2 change in the leucovorin arm relative to placebo, from the model DV ~ Group × Visit + (1|participant) fitted on all 42 thiol substudy participants. q-values are Benjamini–Hochberg adjusted across the five-member thiol family. No metabolite survives correction at $q < 0.05$; the SAM/SAH ratio (nominal $p = 0.020$, $q = 0.098$) and SAH (nominal $p = 0.050$, $q = 0.125$) are reported as nominal findings only. Cohen's d is $\beta / \sqrt{(\sigma^2\_participant + \sigma^2\_residual)}$.

Metabolite	β (Group × Visit)	SE	95% CI	p	q (BH)	Cohen's d
Methionine (µmol/L)	+0.756	0.955	[−1.12, +2.63]	0.429	0.532	+0.25
SAM (nmol/L)	+2.326	2.243	[−2.07, +6.72]	0.300	0.500	+0.36
SAH (nmol/L)	−1.834	0.936	[−3.67, −0.00]	0.050	0.125	−0.67
SAM/SAH ratio	+0.347	0.149	[+0.06, +0.64]	0.020	0.098	+0.77
Homocysteine (µmol/L)	−0.242	0.387	[−1.00, +0.52]	0.532	0.532	−0.24

Supplementary Table S5. Moderation of the thiol and methylation Group × Visit contrast by history of neurodevelopmental regression (Panel A) and by cerebral folate receptor autoantibody status (Panel B).

*Note. Each panel adds one three-way interaction to the thiol model DV ~ Group × Visit × moderator + (1|participant), fitted by REML; β is that three-way coefficient. Panel A uses history of neurodevelopmental regression (NDR) and Panel B uses cerebral folate receptor autoantibody (FRAA) status, each coded so that a negative β denotes a smaller leucovorin-associated increase in the moderator-positive stratum. Benjamini–Hochberg q-values are computed within the five-member thiol family separately in each panel. Cohen's d is β divided by the total standard deviation. Both panels are exploratory: the substudy was not powered for moderation, and no claim rests on a nominal p-value alone. * p < 0.05, ** p < 0.01, *** p < 0.001.*

Metabolite	β (three-way interaction)	SE	p	q (BH)	Cohen's d
**Panel A. Moderation by neurodevelopmental regression (Group × Visit × NDR), n = 42**					
Methionine (μmol/L)	−5.50	1.75	0.0017	0.0085	−1.90
SAM (nmol/L)	−5.68	4.52	0.2090	0.5225	−0.90
SAH (nmol/L)	−1.30	1.88	0.4914	0.5722	−0.47
SAM/SAH ratio	−0.17	0.30	0.5722	0.5722	−0.37
Homocysteine (μmol/L)	+0.64	0.79	0.4174	0.5722	+0.64
**Panel B. Moderation by cerebral folate receptor autoantibody status (Group × Visit × FRAA), n = 42**					
Methionine (μmol/L)	−5.56	1.84	0.0025**	0.0124	−1.85
SAM (nmol/L)	−5.07	4.73	0.2844	0.3555	−0.79
SAH (nmol/L)	+3.03	1.84	0.0999	0.1665	+1.12
SAM/SAH ratio	−0.61	0.29	0.0364*	0.0909	−1.36
Homocysteine (μmol/L)	+0.39	0.81	0.6288	0.6288	+0.40

Methionine subgroup means (μmol/L), by treatment arm and neurodevelopmental regression status

Group	Regression	Visit 1	Visit 2	Δ
Leucovorin	No	19.69	21.18	+1.49
Leucovorin	Yes	23.10	21.42	−1.68
Placebo	No	21.77	19.93	−1.84
Placebo	Yes	19.09	19.58	+0.49

Supplementary Table S6. Concomitant medications at study entry, by treatment arm. Counts are the number of participants using each class at baseline; participants may appear in more than one row. Percentages use the arm denominator. p-values are from Fisher exact tests except the final row, which is a Mann–Whitney U test on the number of classes per participant. Class assignment reflects the granularity of the baseline medication log: antiepileptic agents are recorded as a single class and cannot be separated into valproate and non-valproate agents. Vitamin or dietary supplementation above Recommended Dietary Allowance levels was an exclusion criterion of the parent trial, so no participant was receiving high-dose supplements. No participant was recorded as receiving an immune-modulating agent.

Medication class	Leucovorin n (%)	Placebo n (%)	Total n (%)	p
**Thiol substudy cohort (leucovorin n = 18, placebo n = 24; total n = 42)**				
Stimulants (methylphenidate, amphetamines)	3 (17%)	3 (12%)	6 (14%)	1.000
Melatonin	2 (11%)	4 (17%)	6 (14%)	0.685
Antihistamine / allergy agents	3 (17%)	1 (4%)	4 (10%)	0.297
Gastrointestinal agents	1 (6%)	2 (8%)	3 (7%)	1.000
Alpha-adrenergic agents (guanfacine, clonidine)	2 (11%)	2 (8%)	4 (10%)	1.000
SSRIs / SNRIs	2 (11%)	1 (4%)	3 (7%)	0.567
Antiepileptic drugs	0 (0%)	2 (8%)	2 (5%)	0.498
Antibiotics	2 (11%)	1 (4%)	3 (7%)	0.567
Immune-modulating agents	0 (0%)	0 (0%)	0 (0%)	1.000
Any listed medication	11 (61%)	10 (42%)	21 (50%)	0.350
Mean number of classes per participant	0.83	0.67	—	0.406
**Respirometry cohort (leucovorin n = 17, placebo n = 21; total n = 38)**				
Stimulants (methylphenidate, amphetamines)	3 (18%)	3 (14%)	6 (16%)	1.000
Melatonin	1 (6%)	3 (14%)	4 (11%)	0.613
Antihistamine / allergy agents	3 (18%)	1 (5%)	4 (11%)	0.307
Gastrointestinal agents	1 (6%)	2 (10%)	3 (8%)	1.000
Alpha-adrenergic agents (guanfacine, clonidine)	1 (6%)	2 (10%)	3 (8%)	1.000
SSRIs / SNRIs	2 (12%)	1 (5%)	3 (8%)	0.577
Antiepileptic drugs	0 (0%)	2 (10%)	2 (5%)	0.492
Antibiotics	1 (6%)	1 (5%)	2 (5%)	1.000
Immune-modulating agents	0 (0%)	0 (0%)	0 (0%)	1.000
Any listed medication	9 (53%)	9 (43%)	18 (47%)	0.745
Mean number of classes per participant	0.71	0.71	—	0.859

Medication data were available for all 38 respirometry participants and all 42 thiol substudy participants. No statistically detectable between-arm differences were found for any ascertained class (all $p \geq 0.297$), and the arms did not differ in the proportion using any listed agent or in the mean number of classes per participant. With 17 and 21 participants contributing respirometry, these comparisons have low power and exclude only large imbalances. The log records agent classes rather than individual drugs.

Supplementary Table S6a. Medication-restricted sensitivity analysis of the primary Group × Visit contrast for the respiratory family. Each tier re-fits the pre-specified primary model after excluding participants recorded in Supplementary Table S6 as using the indicated classes. All five respiratory parameters remain significant in every tier, and effect sizes are larger, not smaller, in the medication-free tier.

**Outcome**	**n (leu / pbo)**	**β (Group × Visit)**	**SE**	**p**	**q (BH)**	**Cohen's d**
**Tier 1 — all participants — n = 38 (17 leucovorin / 21 placebo)**						
Basal respiration (BR)	38 (17/21)	+16.66	3.68	<0.001	<0.001	+0.81
ATP-linked respiration (ALR)	38 (17/21)	+12.62	2.63	<0.001	<0.001	+0.84
Proton-leak respiration (PLR)	38 (17/21)	+4.08	1.62	0.012	0.012	+0.48
Maximal respiratory capacity (MRC)	38 (17/21)	+42.81	8.17	<0.001	<0.001	+0.92
Reserve capacity (RC)	38 (17/21)	+25.80	6.74	<0.001	<0.001	+0.71
**Tier 2 — excluding stimulants, alpha-adrenergic agents, SSRIs, antiepileptics — n = 29 (13 leucovorin / 16 placebo)**						
Basal respiration (BR)	29 (13/16)	+17.00	4.19	<0.001	<0.001	+0.80
ATP-linked respiration (ALR)	29 (13/16)	+11.87	3.00	<0.001	<0.001	+0.78
Proton-leak respiration (PLR)	29 (13/16)	+5.13	1.79	0.004	0.004	+0.60
Maximal respiratory capacity (MRC)	29 (13/16)	+41.35	9.62	<0.001	<0.001	+0.84
Reserve capacity (RC)	29 (13/16)	+24.02	7.89	0.002	0.003	+0.64
**Tier 3 — Tier 2 also excluding melatonin — n = 27 (13 leucovorin / 14 placebo)**						
Basal respiration (BR)	27 (13/14)	+17.30	4.30	<0.001	<0.001	+0.81
ATP-linked respiration (ALR)	27 (13/14)	+12.01	3.08	<0.001	<0.001	+0.78
Proton-leak respiration (PLR)	27 (13/14)	+5.20	1.81	0.004	0.004	+0.63
Maximal respiratory capacity (MRC)	27 (13/14)	+41.45	9.84	<0.001	<0.001	+0.85
Reserve capacity (RC)	27 (13/14)	+23.60	8.02	0.003	0.004	+0.65
**Tier 4 — medication-free participants only — n = 20 (8 leucovorin / 12 placebo)**						
Basal respiration (BR)	20 (8/12)	+22.17	5.27	<0.001	<0.001	+0.95
ATP-linked respiration (ALR)	20 (8/12)	+14.94	3.79	<0.001	<0.001	+0.89
Proton-leak respiration (PLR)	20 (8/12)	+7.14	2.22	0.001	0.001	+0.80
Maximal respiratory capacity (MRC)	20 (8/12)	+69.48	11.76	<0.001	<0.001	+1.37
Reserve capacity (RC)	20 (8/12)	+46.80	9.50	<0.001	<0.001	+1.26

Supplementary Table S7. Sensitivity analysis for the primary respiratory and thiol families, restricted to complete-case participants with paired pre- and post-treatment observations.

This sensitivity analysis re-fits the pre-specified primary Group × Visit models on participants contributing both Visit 1 and Visit 2 data. For the respiratory family that is 26 of 38 participants (11 leucovorin, 15 placebo; 1,482 well-level observations). For the thiol family all 42 participants contributed paired plasma once participant identifiers were reconciled, so the complete-case and primary thiol analyses coincide. Effect sizes are modestly attenuated relative to the full respiratory sample but all five parameters remain significant after correction.

Outcome	n	β (SE)	95% CI for β	p	q (BH)	Cohen's d	95% CI for d
**Respiratory family — complete-case participants with paired Visit 1 and Visit 2 data (leucovorin n = 11, placebo n = 15; total n = 26)**							
Basal respiration (BR)	26	+16.38 (4.18)	[+8.18, +24.58]	<0.001	<0.001	+0.75	[+0.37, +1.12]
ATP-linked respiration (ALR)	26	+12.20 (2.92)	[+6.47, +17.93]	<0.001	<0.001	+0.76	[+0.40, +1.12]
Proton-leak respiration (PLR)	26	+4.21 (1.85)	[+0.59, +7.83]	0.023	0.023	+0.46	[+0.06, +0.86]
Maximal respiratory capacity (MRC)	26	+40.74 (9.38)	[+22.36, +59.12]	<0.001	<0.001	+0.82	[+0.45, +1.19]
Reserve capacity (RC)	26	+24.09 (7.68)	[+9.04, +39.14]	0.002	0.002	+0.63	[+0.24, +1.03]
**Thiol / methylation family — all participants had paired Visit 1 and Visit 2 plasma (leucovorin n = 18, placebo n = 24; total n = 42)**							
Methionine (μmol/L)	42	+0.76 (0.95)	[−1.12, +2.63]	0.429	0.532	+0.25	[−0.37, +0.87]
SAM (nmol/L)	42	+2.33 (2.24)	[−2.07, +6.72]	0.300	0.500	+0.36	[−0.32, +1.05]
SAH (nmol/L)	42	−1.83 (0.94)	[−3.67, −0.00]	0.050	0.125	−0.67	[−1.34, −0.00]
SAM/SAH ratio	42	+0.35 (0.15)	[+0.06, +0.64]	0.020	0.098	+0.77	[+0.12, +1.42]
Homocysteine (μmol/L)	42	−0.24 (0.39)	[−1.00, +0.52]	0.532	0.532	−0.24	[−0.99, +0.51]

Note. β is the treatment effect (leucovorin minus placebo in pre-to-post change), so positive values favour leucovorin. q-values are Benjamini–Hochberg adjusted within each outcome family. Cohen's d is $\beta / \sqrt{\sigma^2\_participant + \sigma^2\_residual}$.

Supplementary Table S8. Open-label extension: exploratory single-sequence within-participant phase comparison of the placebo phase (Visit 1 to Visit 2) with the subsequent open-label leucovorin phase (Visit 2 to Visit 3) in participants originally randomised to placebo. Participants originally randomised to placebo received open-label leucovorin between Visit 2 and Visit 3. Because the Visit 2 measurement both ends the placebo phase and begins the leucovorin phase, and because treatment in the extension was open-label and the comparison is confounded by order, it is exploratory and is not a confirmation of the randomised result. The laboratory assays were performed blind to allocation and to phase throughout, so the limitation is one of design rather than of measurement. Only 11 participants contributed respirometry at all three visits and 17 contributed plasma, so strata contain as few as four participants. This is a single-sequence placebo-then-leucovorin design, not an AB/BA crossover, so no sequence or carry-over effect can be estimated. Panel C shows the same interval in participants randomised to leucovorin, who simply continued treatment; only three such participants have respirometry at both Visit 2 and Visit 3, so Panel C cannot serve as a comparator and is shown for completeness.

Outcome	n	Δ (leucovorin phase − placebo phase)	SE / 95% CI	p	Cohen's d	NDR × phase p
**Panel A. Respirometry, paired within-participant contrasts, participant-visit mean across the DMNQ series (n = 11; NDR yes = 7, no = 4)**						
Basal respiration (BR)	11	−2.62	12.64 / −30.78 to +25.54	0.840	−0.06	0.949
ATP-linked respiration (ALR)	11	+4.03	9.26 / −16.60 to +24.67	0.672	+0.13	0.573
Proton-leak respiration (PLR)	11	−6.65	5.22 / −18.30 to +4.99	0.232	−0.38	0.233
Maximal respiratory capacity (MRC)	11	−2.51	27.58 / −63.97 to +58.94	0.929	−0.03	0.524
Reserve capacity (RC)	11	+0.11	21.14 / −46.98 to +47.20	0.996	+0.00	0.379
**Panel B. Thiol / methylation metabolites, paired within-participant contrasts (n = 17; NDR yes = 9, no = 8)**						
Methionine (µmol/L)	17	+1.75	1.00 / −0.38 to +3.88	0.100	+0.42	0.454
SAM (nmol/L)	17	+0.98	3.19 / −5.78 to +7.74	0.762	+0.07	0.756
SAH (nmol/L)	17	−2.03	1.57 / −5.35 to +1.29	0.214	−0.31	0.619
SAM/SAH ratio	17	+0.36	0.21 / −0.08 to +0.81	0.100	+0.42	0.772
Homocysteine (µmol/L)	17	−0.48	0.48 / −1.49 to +0.53	0.324	−0.25	0.284
**Panel C. Group A (leucovorin arm) continuation, Visit 2 to Visit 3 change under continued open-label leucovorin (n = 3) — descriptive only, no inference**						
Basal respiration (BR)	3	−8.42	observed changes −23.44, −16.03, +14.22	not tested	not tested	—
ATP-linked respiration (ALR)	3	−7.36	observed changes −13.01, −12.12, +3.05	not tested	not tested	—
Proton-leak respiration (PLR)	3	−1.05	observed changes −10.42, −3.91, +11.17	not tested	not tested	—
Maximal respiratory capacity (MRC)	3	−20.64	observed changes −40.35, −17.18, −4.40	not tested	not tested	—
Reserve capacity (RC)	3	−12.23	observed changes −31.40, −16.92, +11.63	not tested	not tested	—

Note. Δ is the within-participant difference between the leucovorin-phase change (Visit 3 minus Visit 2) and the placebo-phase change (Visit 2 minus Visit 1), so positive values favour the open-label leucovorin phase. Each participant contributes one value per outcome. The SE and t-based 95% confidence interval are those of the mean paired difference; p is from a one-sample t-test against zero, Cohen's d_z is the mean difference divided by its standard deviation, and the NDR × phase p-value is from a two-sample t-test comparing the paired differences between NDR strata. Panel C rests on three participants, so its observed individual changes are listed and no test statistic is computed. Estimates are computed from participant-level paired differences rather than from individual wells, because wells within a participant-visit are not independent observations and each Visit 2 measurement is shared by both phases. The whole table is exploratory: treatment in the extension was open-label, the leucovorin phase was always second, and period, time and treatment effects are not separable. The assays themselves were performed blind to allocation and phase.

Supplementary Table S9. Exploratory moderation of the primary Group × Visit contrasts by cerebral folate receptor autoantibody (FRAA) status, sex and age. Panel A, Group × Visit × moderator terms for the respiratory outcomes; Panel B, thiol and methylation outcomes; Panel C, the model-estimated arm-specific simple effects underlying the Panel A(i) FRAA coefficients.

Each model adds a three-way Group × Visit × moderator term to the pre-specified primary specification, retaining the DMNQ and DMNQ² dose terms. Reference levels are placebo, Visit 1, FRAA-negative and male; age is continuous in years. A positive coefficient indicates a larger leucovorin-associated increase in the moderator-positive group. Panel A reports the three-way interaction terms only, not the complete model output. Panel C decomposes the Panel A(i) FRAA models into the within-arm pre-to-post change in each FRAA stratum and the within-arm FRAA contrast, evaluated at DMNQ 0; the difference between the two within-arm contrasts equals the Panel A(i) three-way coefficient for each outcome. Panel B applies the same logic to the thiol and methylation family using that family's established specification, DV ~ Group × Visit × moderator with a participant random intercept and no dose terms, since the thiol measurements are single plasma values rather than a DMNQ series; it is fitted on all 42 thiol substudy participants. These analyses are exploratory and limited by small subgroup counts — only 8 girls contributed, 3 of them in the leucovorin arm. Consistent with Section 2.6, Benjamini–Hochberg q-values are reported for the five-member thiol family in Panel B and the respiratory moderator contrasts in Panel A are reported with nominal p-values only.

Note. FRAA status was available for all 38 participants (leucovorin 8 negative / 9 positive; placebo 7 negative / 14 positive). Cohen's d is the three-way coefficient divided by $\sqrt{\sigma^2\_participant + \sigma^2\_residual}$; for age it is expressed per year. In Panel B no moderator term reaches significance for any thiol or methylation outcome; the smallest nominal p-value is 0.072 (Sex × SAH) and the smallest q-value is 0.215, so there is no evidence that the one-carbon findings are moderated by sex or age. Age is taken from the participant-level demographic file of record, which holds a single value per participant.

Outcome	β (three-way term)	SE	p	Cohen's d	q (BH within family)
Panel A(i). Respiratory outcomes — Group × Visit × FRAA-positive (n = 38)					
Basal respiration (BR)	+2.522	3.682	0.493	+0.12	—
ATP-linked respiration (ALR)	+5.029	2.631	0.056	+0.33	—
Proton-leak respiration (PLR)	−2.439	1.620	0.132	−0.28	—
Maximal respiratory capacity (MRC)	+38.287	8.133	<0.001	+0.81	—
Reserve capacity (RC)	+35.725	6.691	<0.001	+0.99	—
Panel A(ii). Respiratory outcomes — Group × Visit × female (n = 38)					

Outcome	β (three-way term)	SE	p	Cohen's d	q (BH within family)
Basal respiration (BR)	−21.262	4.880	<0.001	−1.07	—
ATP-linked respiration (ALR)	−9.918	3.504	0.005	−0.67	—
Proton-leak respiration (PLR)	−11.354	2.141	<0.001	−1.35	—
Maximal respiratory capacity (MRC)	−30.442	10.868	0.005	−0.66	—
Reserve capacity (RC)	−9.598	8.962	0.284	−0.27	—
Panel A(iii). Respiratory outcomes — Group × Visit × age in years (n = 38)					
Basal respiration (BR)	+0.066	0.625	0.916	+0.00	—
ATP-linked respiration (ALR)	+0.415	0.447	0.353	+0.03	—
Proton-leak respiration (PLR)	−0.357	0.275	0.194	−0.04	—
Maximal respiratory capacity (MRC)	+5.485	1.387	<0.001	+0.12	—
Reserve capacity (RC)	+5.378	1.141	<0.001	+0.15	—
Panel B(i). Thiol / methylation outcomes — Group × Visit × female (n = 42)					
Methionine (μmol/L)	−1.073	2.498	0.668	−0.35	0.737
SAM (nmol/L)	−2.000	5.954	0.737	−0.31	0.737
SAH (nmol/L)	+4.170	2.318	0.072	+1.55	0.215
SAM/SAH ratio	−0.643	0.375	0.086	−1.44	0.215
Homocysteine (μmol/L)	+0.427	1.023	0.676	+0.41	0.737
Panel B(ii). Thiol / methylation outcomes — Group × Visit × age in years (n = 42)					
Methionine (μmol/L)	+0.325	0.321	0.311	+0.11	0.456
SAM (nmol/L)	+0.668	0.737	0.364	+0.10	0.456
SAH (nmol/L)	−0.528	0.306	0.084	−0.19	0.225
SAM/SAH ratio	+0.083	0.049	0.090	+0.18	0.225
Homocysteine (μmol/L)	−0.058	0.131	0.659	−0.06	0.659

Panel C. Respiratory outcomes — model-estimated arm-specific simple effects of the Panel A(i) FRAA model, evaluated at DMNQ 0 (n = 38)					
**Arm / FRAA stratum**	**n**	**β (pmol/min)**	**SE**	**95% CI**	**p-value**
Basal respiration (BR)					
Placebo, FRAA negative	7	−7.125	3.035	−13.07 to −1.18	0.019*
Placebo, FRAA positive	14	−4.230	2.789	−9.70 to +1.24	0.129
Placebo, FRAA positive minus negative	21	+2.895	2.545	−2.09 to +7.88	0.255
Leucovorin, FRAA negative	8	+8.347	2.971	+2.52 to +14.17	0.005**
Leucovorin, FRAA positive	9	+13.763	2.858	+8.16 to +19.37	<0.001***
Leucovorin, FRAA positive minus negative	17	+5.416	2.665	+0.19 to +10.64	0.042*
ATP-linked respiration (ALR)					
Placebo, FRAA negative	7	−4.119	2.168	−8.37 to +0.13	0.057
Placebo, FRAA positive	14	−4.427	1.992	−8.33 to −0.52	0.026*

Panel C. Respiratory outcomes — model-estimated arm-specific simple effects of the Panel A(i) FRAA model, evaluated at DMNQ 0 (n = 38)					
**Arm / FRAA stratum**	**n**	**β (pmol/min)**	**SE**	**95% CI**	**p-value**
Placebo, FRAA positive minus negative	21	−0.308	1.817	−3.87 to +3.25	0.865
Leucovorin, FRAA negative	8	+5.744	2.122	+1.58 to +9.90	0.007**
Leucovorin, FRAA positive	9	+10.464	2.041	+6.46 to +14.46	<0.001***
Leucovorin, FRAA positive minus negative	17	+4.720	1.904	+0.99 to +8.45	0.013*
Proton-leak respiration (PLR)					
Placebo, FRAA negative	7	−2.987	1.336	−5.61 to −0.37	0.025*
Placebo, FRAA positive	14	+0.134	1.229	−2.28 to +2.54	0.913
Placebo, FRAA positive minus negative	21	+3.121	1.122	+0.92 to +5.32	0.005**
Leucovorin, FRAA negative	8	+2.601	1.308	+0.04 to +5.17	0.047*
Leucovorin, FRAA positive	9	+3.283	1.259	+0.81 to +5.75	0.009**
Leucovorin, FRAA positive minus negative	17	+0.681	1.172	−1.62 to +2.98	0.561
Maximal respiratory capacity (MRC)					
Placebo, FRAA negative	7	−4.872	6.703	−18.01 to +8.26	0.467
Placebo, FRAA positive	14	−15.200	6.158	−27.27 to −3.13	0.014*
Placebo, FRAA positive minus negative	21	−10.328	5.616	−21.34 to +0.68	0.066
Leucovorin, FRAA negative	8	+16.447	6.561	+3.59 to +29.31	0.012*
Leucovorin, FRAA positive	9	+44.406	6.310	+32.04 to +56.77	<0.001***
Leucovorin, FRAA positive minus negative	17	+27.959	5.887	+16.42 to +39.50	<0.001***
Reserve capacity (RC)					
Placebo, FRAA negative	7	+2.568	5.520	−8.25 to +13.39	0.642
Placebo, FRAA positive	14	−10.804	5.076	−20.75 to −0.85	0.033*
Placebo, FRAA positive minus negative	21	−13.372	4.617	−22.42 to −4.32	0.004**
Leucovorin, FRAA negative	8	+8.134	5.406	−2.46 to +18.73	0.132
Leucovorin, FRAA positive	9	+30.487	5.202	+20.29 to +40.68	<0.001***
Leucovorin, FRAA positive minus negative	17	+22.353	4.843	+12.86 to +31.85	<0.001***
